# The Loricrin-Like Protein (LLP) of *Phytophthora infestans* Is Required for Oospore Formation and Plant Infection

**DOI:** 10.3389/fpls.2017.00142

**Published:** 2017-02-09

**Authors:** Ting Guo, Xiao-Wen Wang, Kun Shan, Wenxian Sun, Li-Yun Guo

**Affiliations:** Department of Plant Pathology and the Key Laboratory for Plant Pathology MOA, China Agricultural UniversityBeijing, China

**Keywords:** sexual reproduction, oxidative stress, reactive oxygen species, pathogenicity, *Phytophthora infestans*, DPI

## Abstract

Loricrin-like protein (LLP) is characterized by a high content of glycine residues and is a major component of plant cell wall. Here, we identified a *Phytophthora infestans* ortholog of plant *LLP*, named *PiLLP*. In *P. infestans, PiLLP* is strongly expressed in asexual and sexual developmental stages, including in sporangia, zoospores and germinating cysts, and during oospore formation, as well as in the early stages of infection and during hydrogen peroxide stress. Compared with the wild type, the *PiLLP*-silenced transformants were defective in oospore formation, had slower colony expansion rates, produced less sporangia with lower germination and zoospore-release rates, and were more sensitive to hydrogen peroxide. Moreover, Nile red staining, and *PiLLP*-red fluorescent protein fusions indicated that PiLLP is involved in oogonia formation. The silenced transformants also had severely diminished virulence levels that could be partially restored with diphenyleneiodium treatments. The analysis of catalase activity showed a decrease of catalase activity in silenced transformants. Thus, *PiLLP* is important for sexual and asexual reproduction, and is required for oxidative stress tolerance and plant infection.

## Introduction

The oomycetes form a group of fungus-like eukaryotes containing diverse microorganisms that live in saltwater, freshwater, and terrestrial environments (Sparrow, 1960; Karling, 1981). A phylogenetic analysis showed that oomycetes belong to the Stramenopila kingdom, along with photosynthetic chromistan algae (Sogin and Silberman, 1998; Baldauf et al., 2000; Yoon et al., 2002; Adl et al., 2005). Many members of the oomycetes are well-known pathogens of plants and animals, causing severe economic losses. *Phytophthora infestans* (Mont.) de Bary is the causal agent of potato late blight, which is a devastating disease worldwide and the causal agent of the Irish potato famine of the Nineteenth century (Bourke, 1993).

*P. infestans* reproduces asexually by forming sporangia on sporangiophores. Sporangia are readily dehiscent, particularly in response to changes in relative humidity (Aylor et al., 2001). Sporangia in free water germinate directly either via germ tubes at higher temperatures (20–25°C), or by releasing zoospores at lower temperatures (10–15°C) (Ribeiro, 1983). The asexual cycle enables dramatically rapid population growth in susceptible host tissue. Both sporangia and zoospores are important for infection and enable the movement of *P. infestans* between host plants.

As a heterothallic oomycete, *P. infestans* requires the presence of both mating types, as in “MX5-1” (A1 mating type) and “80787-94L” (A2 mating type), to complete sexual reproduction by producing oospores. The thick-walled oospores can overcome harsh environmental conditions, such as cold, chemical fumigation, and microbial degradation, surviving in soil or plant debris for many years. They then serve as primary inocula in the following years (Mayton et al., 2000; Turkensteen et al., 2000; Prakob and Judelson, 2007). Moreover, the gene recombination resulting from sexual reproduction may generate new virulent strains making disease management more difficult (Gavino et al., 2000). Although some genes involved in the sexual reproduction of *Phytophthora* have been screened (Fabritius et al., 2002; Prakob and Judelson, 2007; Zhao et al., 2011), the biological functions of these genes have not been explored.

Generally, *P. infestans* is regarded as a hemi-biotrophic pathogen that displays host specificity. Many pathogenicity factors have been identified to be essential for successful infection and colonization of the host, including the cytoplasmic RXLR effector Avr3a (Sanju et al., 2015), the bZIP transcription factors (Gamboa-Meléndez et al., 2013), the carbohydrate-active enzymes (Brouwer et al., 2014) and endopolygalacturonase (Torto et al., 2002).

During evolution, plants developed a sophisticated defense system to counter microbial invasions. Upon infection, plants defend themselves through biochemical reactions, such as the accumulation of reactive oxygen species (ROS) at the plant surface (Apostol et al., 1989). The production of ROS is catalyzed by nicotinamide adenine dinucleotide phosphate (NADPH) oxidases that are localized in the plasma membrane (Doke et al., 1996). To successfully infect the host plant, pathogens have to scavenge the plant-derived ROS (Apel and Hirt, 2004). Recently, effectors, mitogen-activated protein kinases (MAPKs), heat-shock proteins and bZIP transcription factors in *Phytophthora* spp. have been proven to be involved in ROS scavenging (Dong et al., 2011; Gamboa-Meléndez et al., 2013; Gao et al., 2015; Sheng et al., 2015). In total, five catalase genes are encoded in the genome of *P. infestans*. A transcriptome analysis of *P. infestans* has revealed that catalase genes (*PITG_15248* and *PITG_07143*) were up-regulated during early infection stages (Haas et al., 2009). Thus, ROS scavenging maybe important for *P. infestans* pathogenesis.

Loricrin is a type of glycine-, serine-, and cysteine-rich protein found in animals and is a major component of the cornified cell envelope (CE) in terminally differentiating structures (Hohl et al., 1993). Its major function is to strengthen the CE and the defensive barrier (Nithya et al., 2015). The glycine-rich proteins (GRPs) are loricrin-like proteins (LLPs) found in plants (Goddemeier et al., 1998). GRP is a major component of plant cell walls (Cassab, 1998) and is speculated to play important roles in the development of vascular tissues, nodules and flowers (Ryser and Keller, 1992; de Oliveira et al., 1993; Küster et al., 1995; Ryser et al., 1997). Currently, in plants, five classes of GRPs have been defined based on the arrangement of the glycine repeats and the type of conserved motifs (Mangeon et al., 2010, 2016). The Class I GRPs have a high glycine-content region of (GGX)_n_ repeats, such as the French bean PvGRP1.8, which performs a structural role in the cell wall as a part of the protoxylem repair system (Ringli et al., 2001). The Class II GRPs contain a characteristic cysteine-rich C-terminus, as seen in NtCIG1 in tobacco, which enhances the callose deposition in cells (Ueki and Citovsky, 2002). The Class III GRPs have an oleosin domain, like that in AtOGB3, which is required for pollen hydration and competition (Mayfield and Preuss, 2000). Class IV GRPs are also known as RNA-binding GRPs, like AtCSG2, which is implicated in cold, salt, and osmotic stresses (Park et al., 2009). The Class V GRPs, containing mixed patterns of repeats, have been identified in eucalyptus (Bocca et al., 2005), but their functions are unknown.

By mining the microarray data obtained by Prakob and Judelson (2007), we found a novel gene encoding a LLP, which has not been studied before. As it was highly expressed during the sexual development of *P. infestans*, we suspected it played an important role in oospore formation. Here, we investigated the biological roles of this LLP gene in *P. infestans* using gene silencing and over-expression techniques. Our study provides insights into the *LLP*'s function during oospore formation and the pathogenesis of oomycetes.

## Materials and methods

### Phylogenetic analysis

Gene sequences of oomycete species used in this study were retrieved from the database of the DOE Joint Genome Institute (http://www.jgi.doe.gov/) and the EnsemblGenomes (http://www.ensemblgenomes.org/). Based on additional searches genes of diatoms (*Thalassiosira pseudonana*), protists (*Tetrahymena thermophile, Paramecium tetraurelia*), and plants (*Arabidopsis thaliana, Sorhum bicolor, Zea mays, Oryza sativa*) were retrieved from the NCBI and KEGG databases (Table S1). Multiple alignments of the amino acid sequences were performed using ClustalW2 (http://www.ebi.ac.uk/Tools/msa/clustalw2/; Larkin et al., 2007). Phylogenetic trees were constructed with the maximum likelihood evolution algorithm in MEGA 5.22 (Tamura et al., 2011). A Poisson correction was used for multiple substitution models and pairwise deletion was used for gap split data treatment. The statistical strengths were assessed by bootstraps with 1000 replicates.

### *P. infestans* strains and culture conditions

The “MX5-1” (A1 mating type; Han et al., 2012) and “80787-94L” (A2 mating type; Guo et al., 2010) isolates of *P. infestans* were used in this study. The WT strain and silenced transformants were maintained on tomato rye agar (TRA) medium (Guo et al., 2010) at 18°C in the darkness. Oospores were obtained by pairing “MX5-1” (A1 mating type) and “80787-94L” (A2 mating type) on TRA plates kept in the darkness for 7, 11, and 17 days. To test the mycelial growth rate, culture blocks (5 mm in diameter) cut from the edge of an actively growing culture were inoculated on the TRA plate at 18°C in the darkness, and the colony diameters were measured after a 7-day incubation. Sporangia of each strain were collected by washing 12-day-old cultures with pea broth. Zoospores were generated by incubating the sporangia suspension first at 4°C for 2 h and then at room temperature for 1 h. To test the oxidative sensitivity of each strain, culture blocks (5 mm in diameter) were placed on fresh TRA plates supplemented with 0.5, 1.0, 1.5, and 2.0 mM H_2_O_2_. The colony diameters were then measured after incubating the culture at 18°C in the darkness for 7 days. All of the experiments were performed twice, and each treatment was repeated in triplicate.

### RNA extraction and gene expression analysis

To determine the transcript profiles of the PiLLP gene in various stages of sexual development, the “MX5-1” (A1 mating type) and “80787-94L” (A2 mating type) isolates were incubated 2-cm apart from each other on polycarbonate membranes with 0.4 μm pores (Merck Millipore Ltd., Tullagreen, Ireland) overlaid on the surface of TRA plates and incubated at 18°C in the darkness. Each of these isolates, paired similarly with itself and cultured under the same conditions, was used as control. After 4, 6, 8, 10, 12, and 14 days, mycelia, and mycelia with oospores, were harvested from the junction area between the inoculation sites in each plate and were then ground in liquid nitrogen. The total RNA of each sample was extracted using a NucleoSpin RNA Plant kit (MACHEREY-NAGEL, Düren, Germany), and was reversely transcribed with an oligo (dT)_18_ primer using the Reverse Transcriptase M-MLV (TaKaRa Bio Inc. Shiga, Japan) following the manufacturer's instructions. SYBR Green real-time qPCRs were performed in an ABI 7500 detection system (Applied Biosystems, Foster City, CA, USA). The elongation factor 1 (*ef1*) gene of *P. infestans* was used as an internal control. Means and standard deviations were calculated using data from three replicates. The whole experiment was repeated with a different set of biological samples.

### Plasmid construction and the generation of *P. infestans* transformants

We used the plasmid vector pTORmRFP4 to construct transformation plasmids. The 5′UTR, 3′UTR, and ORF regions of *PiLLP* were amplified from the cDNA of *P. infestans* “MX5-1” with specific primers (Table S2). The amplified fragments of 5′UTR and 3′UTR were joined by fusion PCR, and then inserted in an anti-sense orientation into pTORmRFP4 for silencing. The ORF of *PiLLP* was inserted in the sense orientation for overexpression. The plasmid constructs were all verified by DNA sequencing. Transformation of the *P. infestans* strain “MX5-1” was performed as described previously (Mcleod et al., 2008). To validate the transformants, *PiLLP* transcript levels during the growth of mycelia were measured by qRT-PCR.

### Tetrazolium bromide staining

Oospores from STs (*PiLLP*-silenced transformants), CK (the transformant carrying the empty vector pTORmRFP4) and WT (MX5-1) strains were obtained as described above. MTT staining was performed by mixing the oospore suspension with equivalent volumes of 0.1% MTT (Sigma-Aldrich, St. Louis, MO, USA) in 0.1 M PBS (pH 5.8), and incubated at 37°C for 24 h before observing under an Olympus BX41 microscope (Olympus, Tokyo, Japan). The oospores in red or purple were considered to be dormant viable, whereas the black and unstained oospores were non-viable (Ribeiro, 1978).

### Nile red staining

The distribution of lipid bodies in oospores was determined using Nile red staining. The Nile red stock solution (1 mM) was prepared by dissolving 100 mg of Nile red powder (Sigma-Aldrich, St. Louis, MO, USA) in 314 ml of methanol. Before staining, 10 μl of stock solutions were diluted 20 × with dimethylsulphoxide (DMSO). An aliquot of 20 μl oospore suspension was mixed with an equal volume of diluted Nile red solution on a microscope slide, and incubated at room temperature for 5 min, followed by two washes with PBS (pH 5.8). The samples were then viewed under an Olympus FluoView™ FV1000 confocal laser scanning microscope (Olympus, Tokyo, Japan) with an excitation wavelength of 530 nm and an emission wavelength of 568 nm.

### Trypan blue staining

Trypan blue staining was performed as described by Koch and Slusarenko (1990). Infected leaves were stained with lactophenol-trypan blue (10 ml of lactic acid, 10 ml of glycerol, 10 g of phenol, and 10 mg of trypan blue dissolved in 10 ml of distilled water). The leaves were boiled for ~2 min in the staining solution and then destained in 2.5 g/ml chloral hydrate for 30–40 min.

### 3, 3-diaminobenzidine (DAB) staining

ROS were stained with the oxidant-sensitive probe DAB (Sigma-Aldrich, St. Louis, MO, USA) as described by Liu and Friesen (2012). Detached *Nicotiana benthamiana* leaves were inoculated with the sporangia of WT, CK, and transformants. After 12 h, the leaves were incubated in 1 mg/ml DAB solution (pH 3.8) at room temperature for 8 h, and were destained with ethanol, followed by viewing using an Olympus Bx41 microscope (Olympus, Tokyo, Japan).

### Pathogenicity test

For plant inoculations, WT (MX5-1), CK, STs, and OTs (*PiLLP*-overexpressed transformants) strains were incubated on TRA medium- plates in the darkness for 14 days. Sporangia of each strain were collected by washing 12-day-old cultures with pea broth. A 20-μl droplet of a sporangial suspension of 1 × 10^4^ sporangia/ml for WT, CK, and OTs and 2 × 10^4^ sporangia/ml for ST strains was inoculated on a detached potato (cv. Impala) leaf. Three detached leaves placed on a moist filter paper in a Petri dish were used as a replicate, and three plates were used for each treatment. Inoculated leaves were then incubated at 21°C with a 12-h/12-h light/ dark cycle for 5 days. The size of each lesion was measured and analyzed with Student's *t*-test. The experiments were performed twice.

### DPI infiltration

DPI, a flavoenzyme inhibitor, inhibits the activities of the plant NADPH oxidases that are necessary for ROS generation (Cross and Jones, 1986). To scavenge the host-derived ROS, leaves of potato (cv. Impala) were infiltrated with 0.5 μM DPI (Sigma-Aldrich, St. Louis, MO, USA) in 0.05% DMSO using a disposable, plastic needleless syringe. The leaves that were infiltrated with 0.05% DMSO served as controls (Eloy et al., 2015).

### Electron microscopy

We used scanning electron microscopy (SEM) to observe the surfaces of oogonial walls in WT, CK and ST strains. Oospores were harvested as described early from 11-day-old cultures grown on TRA medium, and samples were prepared as described by Ho (1979). Images were obtained using a HitachiS-3400N (Hitachi, Tokyo, Japan).

### In-gel assay for activity of catalase

In order to investigate the catalase activity of WT, CK, and transformants, mycelia cultured in Pea Broth (200 g/L) for 4 days were collected through filtration. Each sample was ground in liquid nitrogen and then mixed with ice-cold extraction buffer containing 50 mM HEPES (pH7.4), 137 mM NaCl, 10% Glycerol and protease inhibitors, Pepstatin A (1 μg/mL), Leupeptin (1 μg/mL), PMSF (1 mM). Mixtures were centrifuged at 10,000 g for 10 min at 4°C. Supernatant was collected and used as crude extract (Wang et al., 2016). The protein concentration was calculated based on the absorbance at 595 nm with Bio-Rad protein assay dye.

In-gel assay was performed as described previously (Lledıas et al., 1998). Equal amounts of total protein (20 μg) of different strains (WT, CK, STs, and OTs) were loaded into a 7.5% native polyacrylamide slab gel. The gel was immersed in 7 mM H_2_O_2_ for 10 min after electrophoresis, and then incubated in a 1/1 mixture of freshly prepared 1% potassium hexacyanoferrate (III) and 1% iron (III) chloride hexahydrate. Blue color was observed in the gel except at zones where H_2_O_2_ was decomposed by catalases (Harris and Hopkinson, 1987).

## Results

### *LLP* is a conserved gene in oomycetes

**Figure 1:**
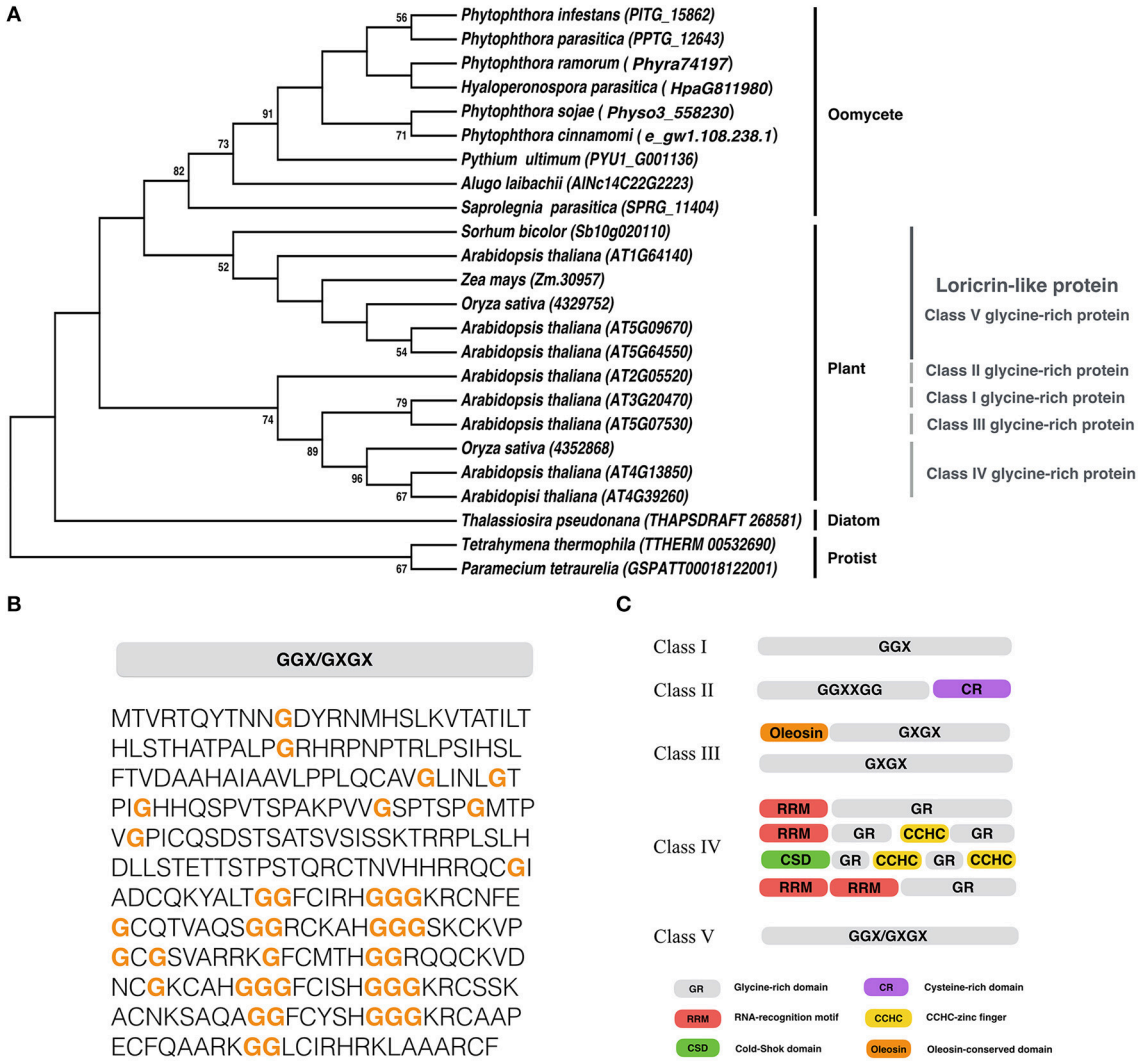
**Sequence analysis of ***PiLLP***. (A)** Phylogenetic analysis of LLP from different organisms. A maximum-likelihood tree was constructed using MEGA 5 with LLP sequences from the species including, *Phytophthora cinannmomi, Phytophthora infestans, Phytophthora ramorum, Phytophthora sojae, Phytophthora parasitica, Albugo laibachii, Hyaloperonospora parasitica, Pythium ultimum, Saprolegnia parasitica, Thalassiosira pseudonana, Micromonas pusilla, Arabidopsis thaliana, Oryza sativa, Sorhum bicolor, Zea mays, Paramecium tetraurella*, and *Tetrahymena thermophile*. Phylogenetic trees were constructed with the maximum likelihood evolution algorithm in MEGA 5.22. The statistical strengths were assessed by bootstraps with 1000 replicates. Bootstrap values (≥50%) are shown near the tree nodes. **(B)** The complete amino acid sequence of PiLLP (accession number XP_002898099). Orange bold letter, glycine repeat region. **(C)** Schematic representation of plant glycine-rich proteins classification. CCHC, CCHC-zinc finger; CSD, Cold-shock domain; CR, cysteine-rich domain; GR, Glycine rich domain; Oleosin, Oleosin-conserved domain; RRM, RNA-recognition motif. Glycine-rich repeats are indicated as GGX, GGXXXGG, GXGX, and GGX/GXGX, where G represents glycine and X represents any amino acid (Modified from Mangeon et al., 2010).

We identified a gene encoding LLP in *P. infestans*, and designated it as *PiLLP* (http://protists.ensembl.org/Phytophthora_infestans/Gene/Compara_Tree?anc=1452626;db=core;g=PITG_15862;g1=BN9_002120;r=supercont1.39:614858-615784;t=PITG_15862T0, gene ID: PITG_15862). The *PiLLP* gene is 927 bp in length and encodes a protein of 308 amino acids (aa) with a glycine content of 12.7%. Only one homolog of *PiLLP* was found in each oomycete genome, and a phylogenetic analysis showed that all of the oomycete LLPs were grouped into one clade, clustering with the Class V GRPs in plants (Figure 1A). A structural analysis showed that these LLPs contain mixed patterns of GGX/GXGX repeats, similar to the plant Class V GRPs (Figures 1B,C). However, the plant Class V GRPs are larger, around 600 aa, so almost double the size of the oomycete LPPs. These results suggest that the LLP genes are highly conserved in oomycetes and are phylogenetically related to the Class V GRPs of plants. No orthologs of oomycete or plant LLPs were found in fungi.

### The expression pattern of *PiLLP* in different stages

**Figure 2:**
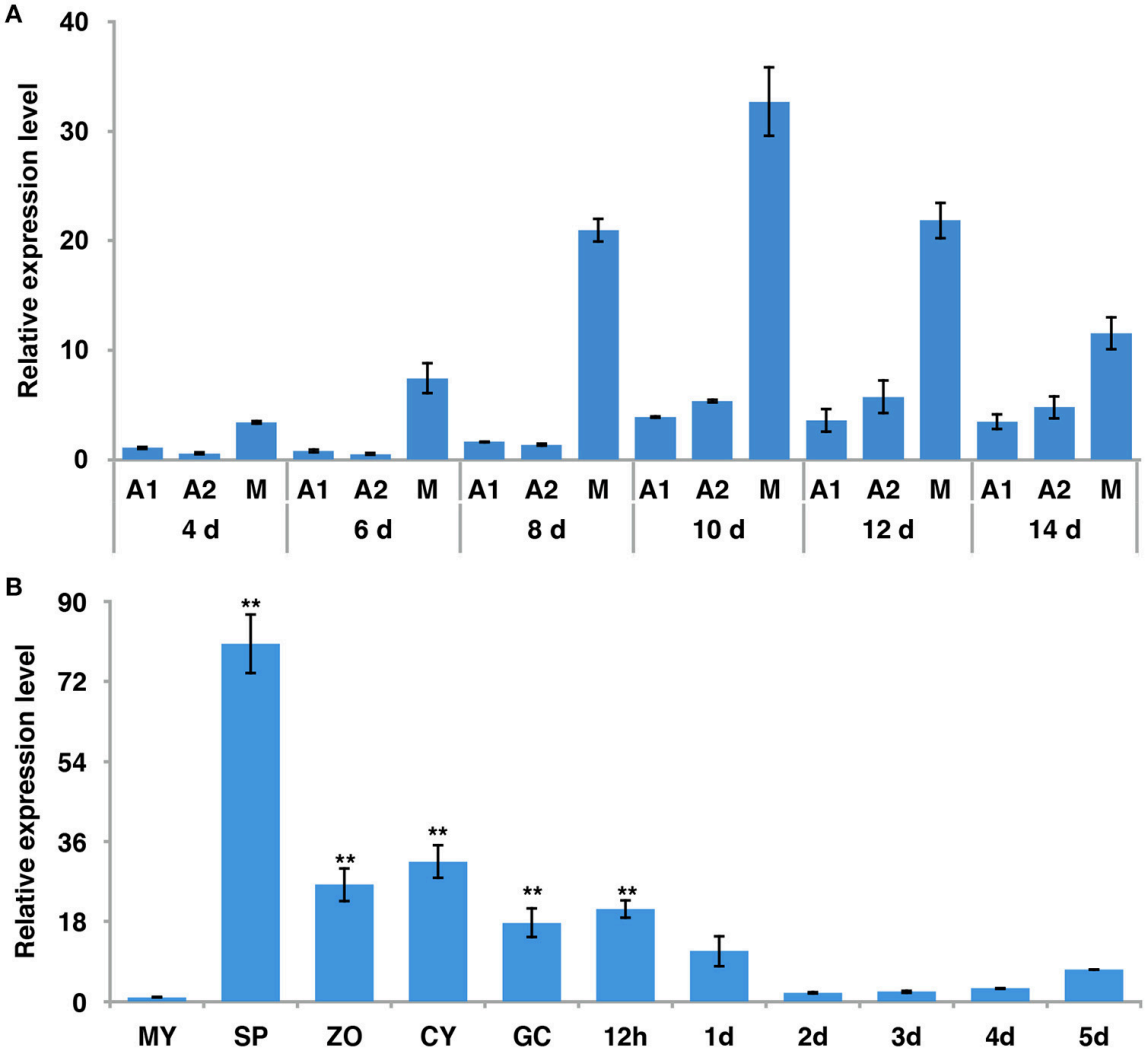
**The expression of ***PiLLP*** detected using qRT-PCR. (A)** *PiLLP* expression in 4, 6, 8, 10, 12, and 14 days cultures with 4 days cultures of A1 mating type(MX5-1) as a reference (value, 1.0). **(B)** *PiLLP* expression in vegetative mycelia (MY), sporangia (SP), zoospores (ZO), cysts (CY), cyst germination (GC), and in the susceptible patato cv. Impala at different time course post-inoculation, in comparison to MY (value, 1.0). The elongation factor 1 *(ef1)* gene was used as an inner control. Three replicates were used for each treatment and the whole experiment was repeated with a different set of biological samples. Bars represent the standard deviation of three replicates. Statistical significance was analyzed using Student's *t*-test. Two asterisks indicate the significant difference (^**^P < 0.01).

To examine the expression pattern of *PiLLP*, we analyzed the transcript levels in *P. infestans* during sexual development (mating), asexual development (mycelia, sporangia, zoospores, cysts, and germinated cysts) and in planta growth (infection stages) using real-time qPCR. During mating, the *PiLLP* transcript level was up-regulated from 4 to 14 day, with the highest expression value at 10 day (Figure 2A; Table S4), and the *PiLLP* transcript level in sporangia was 80-fold that in mycelia (Figure 2B; Table S4). During infection, the *PiLLP* expression was highest at 12-h post-inoculation, then dropped and maintained a low level from 1-day post-inoculation (Figure 2B; Table S4). Thus, *PiLLP* may play important roles in reproduction and plant infection of *P. infestans*.

### Generation of *PiLLP* transformants

**Figure 3:**
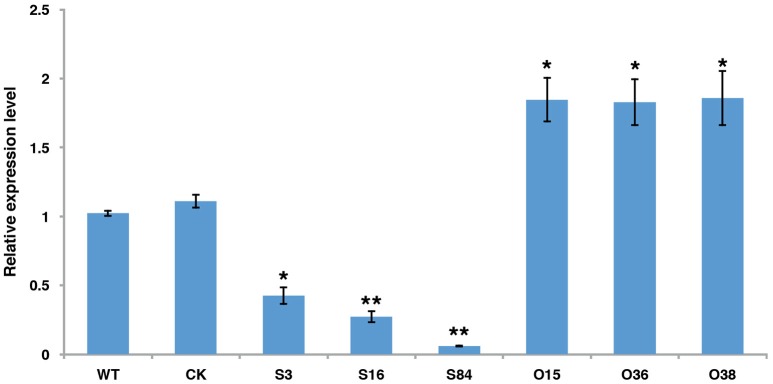
**The expression levels of ***PiLLP*** in WT, CK, and the ***PiLLP*** transformants (S3, S16, S84, O15, O36, and O38)**. qRT-PCR assay was performed using cDNAs synthesized from mycelial RNAs of different isolates. The level of *PiLLP* expression was determined relative to that of *ef1*, and WT was used as a reference (value, 1.0). Three replicates were used for each treatment and the whole experiment was repeated with a different set of biological samples. Bars represent the standard deviation of three replicates. Statistical significance was analyzed using Student's *t*-test between WT and each transformant (^*^P < 0.05, ^**^P < 0.01).

To investigate the function of *PiLLP, PiLLP*-silenced transformants (STs) and *PiLLP*-overexpressed transformants (OTs) were generated by polyethylene glycol-mediated protoplast transformation. The STs were generated with pTORmRFP4 vectors containing 5′ and 3′ untranslated regions (UTR) of *PiLLP*, whereas the OTs were generated with pTORmRFP4 vectors containing the *PiLLP* open reading frame (ORF) region. A transformant harboring the empty vector pTORmRFP4 was used as the control (CK). In total, three STs (S3, S16, and S84), three OTs (O15, O36, and O38), and a CK strain were used in this study. The expression levels of *PiLLP* in STs were from 10 to 45% of that in the WT strain, whereas the values in the OTs were significantly higher than those of WT and CK strains (Figure 3; Table S4).

### Sexual phenotypes of *PiLLP*-silenced transformants

**Figure 4:**
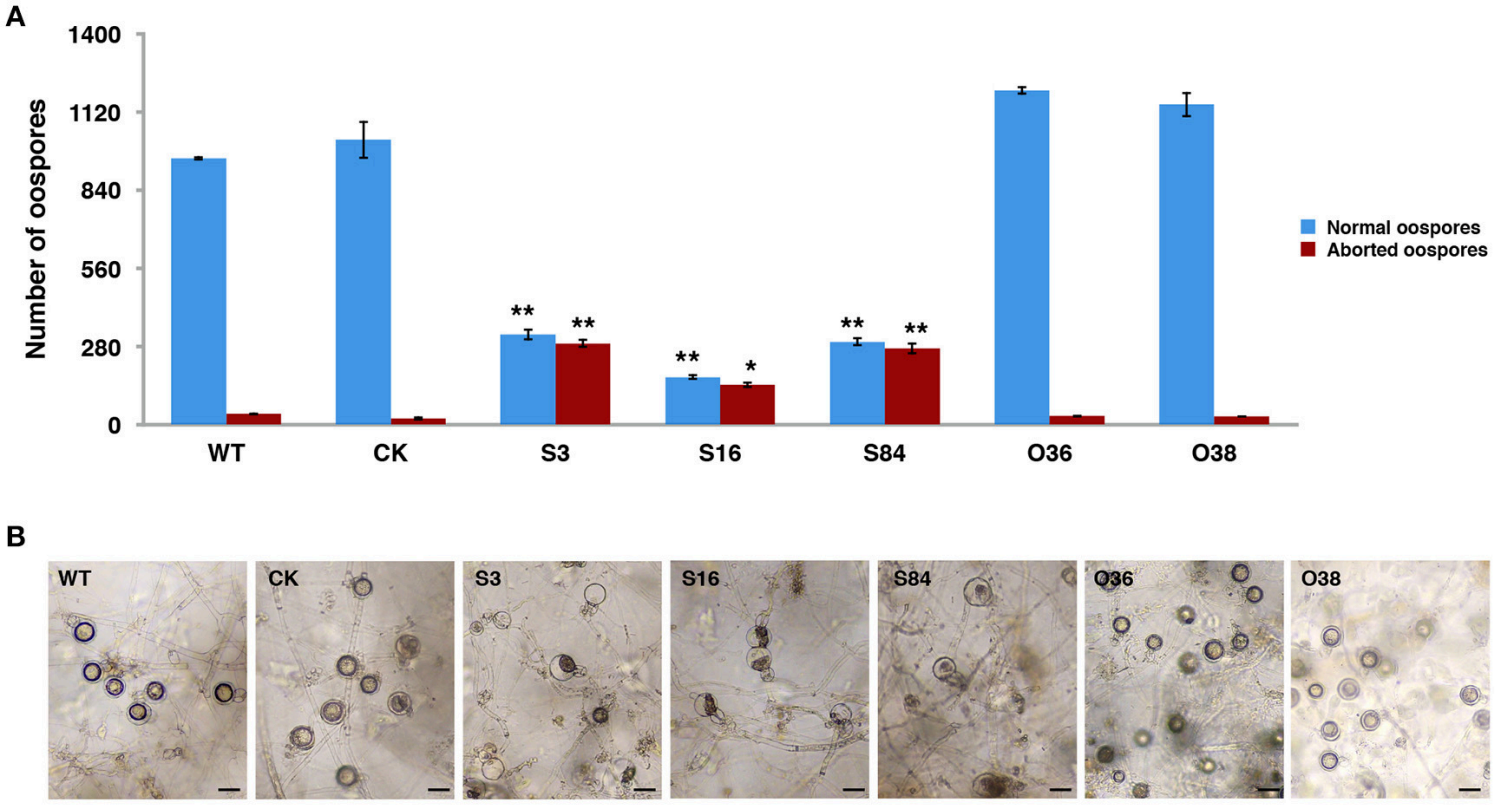
*****PiLLP***-silenced transformants produced many aborted oospores. (A)** Amount of normal and aborted oospores produced by wild-type (WT), control strain (CK, carrying the empty vector of pToRmRFP4), and the *PiLLP*-silenced transformants (S3, S16, and S84). **(B)** Morphology of oospores produced in 17 days old cultures of WT, CK, and *PiLLP*-silenced transformants (S3, S16, and S84). The bars indicate 20 μm. ^*^P < 0.05, ^**^P < 0.01.

All of the transformants, WT and CK were mated with the A2 mating-type isolate (80787-94L) on TRA medium to produce oospores. Less oospores were formed in paired-cultures of STs in comparison with the WT, CK, and OTs, and ~45% (Table S3) of the oogonia in these ST strains did not form thick-walled oospores, whereas this was only ~4% in WT, CK and OT cultures (Figures 4A,B).

**Figure 5:**
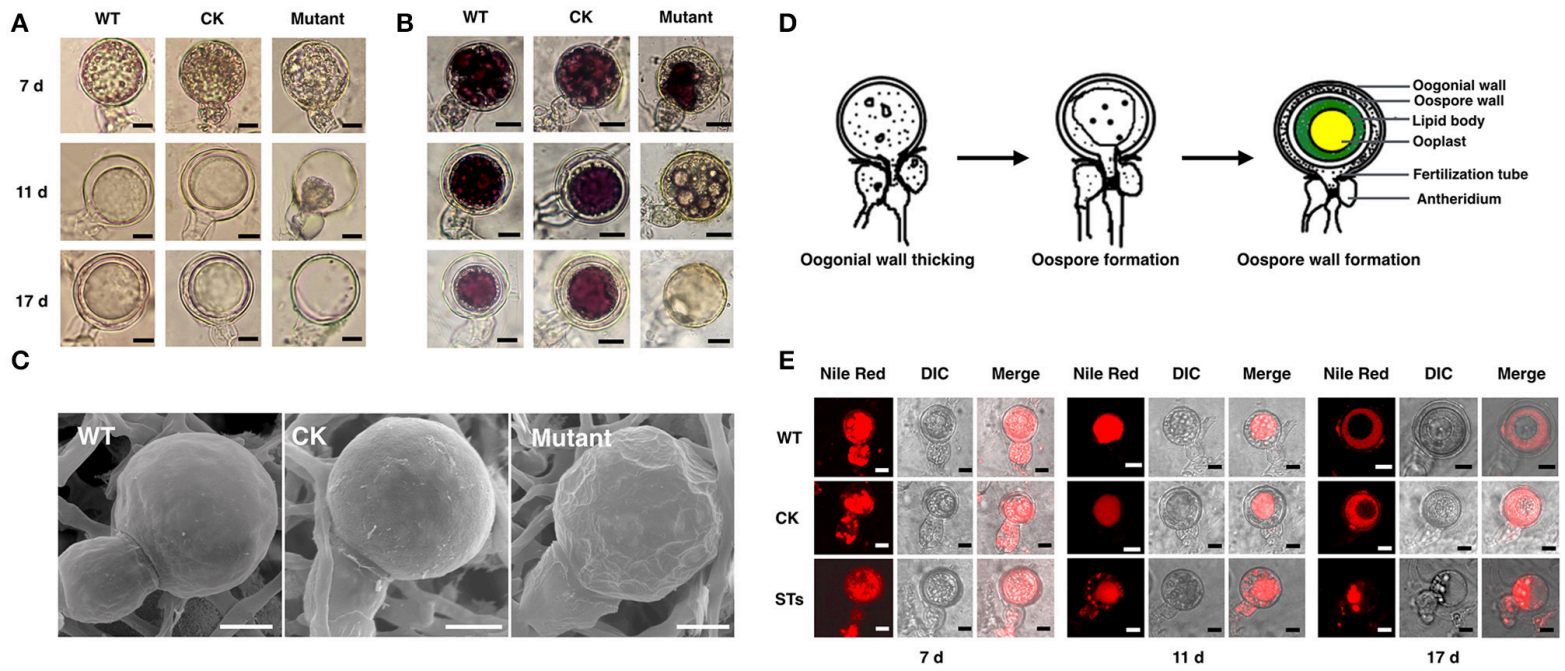
**The morphology of oospores at different developing stages**. The oospores from WT, CK, and *PiLLP*-silenced transformants (STs) were observed with light microscope before **(A)** or after **(B)** they were treated with tetrazolium bromide (MTT), or were observed with Scanning Electronic Microscope **(C)**. Oospores from 7, 11, and 17 days old cultures were used and those stained red were considered to be viable, whereas the unstained ones were nonviable. Oospores in **(C)** were from 11 days old cultures. The bars indicate 10 μm. **(D)** Development of oospores. At the early stage of oospore development, oogonial wall begin to thick, and then oospore was formed, finally the mature oospore was produced, consisting of oospore wall, lipid body and ooplast (Drawing according to Hemmes, 1983). **(E)** The distribution of lipid body in oogonia at different developing stages. Oogonia from 7, 11, and 17 days old cultures of WT, CK, and *PiLLP*-silenced transformants (STs) were treated with Nile red and observed under fluorescence microscope. The red emission indicated the distribution of lipid body. The bars indicate 10 μm.

To investigate the effects of *PiLLP*-silencing on oospore development, sexual apparatuses (oogonia–antheridia) formed in 7-, 11-, and 17-day-old cultures were investigated. Because the same A2 mating-type isolate (80787-94L) was used in all the pairings, the oogonia were referred as 7-, 11-, and 17-days-old for convenience. No obvious difference was found among the 7-day-old oogonia of different strains (Figure 5A). However, about half of the 11-day-old oogonia of the STs showed a decline in the amount of cytoplasm in oogonia compared with those of the WT and CK strains at the same stage (Figure 5A). Moreover, the cytoplasm in ~45% of the 17-day-old oogonia of the STs was completely void, without oospores, whereas the majority of the oogonia of WT and CK strains at the same stage contained mature thick-walled oospores (Figure 5A).

Subsequently, we determined the viability of oospores through tetrazolium bromide (MTT) staining. The 7-, 11-, and 17-day-old oospores of WT and CK strains, and the 7-day-old oogonia of the STs were stained purple (Figure 5B), indicating that they are viable. However, nearly half of the 11- and 17-day-old oogonia in the STs were black or unstained, suggesting the loss of viability (Figure 5B). Thus, some STs could form viable immature oogonia, but others could not form oospores (Figure 5B). When the 11-day-old oogonia from STs, WT, and CK strains were observed by SEM, smooth oogonial walls appeared in the oogonia of WT and CK, whereas many sunken areas were observed on the surfaces of ~50% of the oogonia of the STs, indicating the loss of turgor in these oogonia (Figure 5C).

A previous study showed that oogonia of *Phytophthora* went through the following three distinct stages during the oogonial expansion phase: (1) the oogonial wall thickens from 200 nm to 800 nm; (2) the largely “functional” cytoplasm of the oogonium changes to vacuoles and lipid bodies by the time the oosphere is formed; (3) the oospore forms and the central ooplast is surrounded by a cortex of lipid bodies (Hemmes, 1983; Figure 5D). We stained the developing oogonia with Nile red to explore in which stage development is blocked. As shown in Figure 5E, the 7-day-old oogonia of STs exhibited the same phenotypes as those of the WT and the CK strains, emitting red fluorescence both in oogonia and in antheridia, indicating that lipid bodies were present in both. Almost all the 11-day-old oogonia of WT and CK showed red fluorescence only in oogonia, indicating that the lipid body in the antheridium is either degenerated or translocated into the oogonium, while the substance with red fluorescence was aggregated to form a “red ball.” This kind of “red ball” was then observed in almost all of the oospores formed in the 17-day-old oogonia of WT and CK strains, while a little red fluorescence remained in the area between the wall of the oospore and the oogonium. However, nearly half of the 11-day-old oogonia of STs showed red fluorescence both in oogonia and in antheridia, and the red fluorescence-emitting substance was scattered instead of aggregating into a red ball (Figure 5E). In the 17-day-old oogonia of STs, no mature oospores were formed and only a small amount of red fluorescence-emitting substance was left in oogonia and antheridia (Figure 5E). Thus, the sexual development in STs is blocked in the stage of oospore wall formation.

### Asexual phenotypes of *PiLLP* transformants

**Figure 6:**
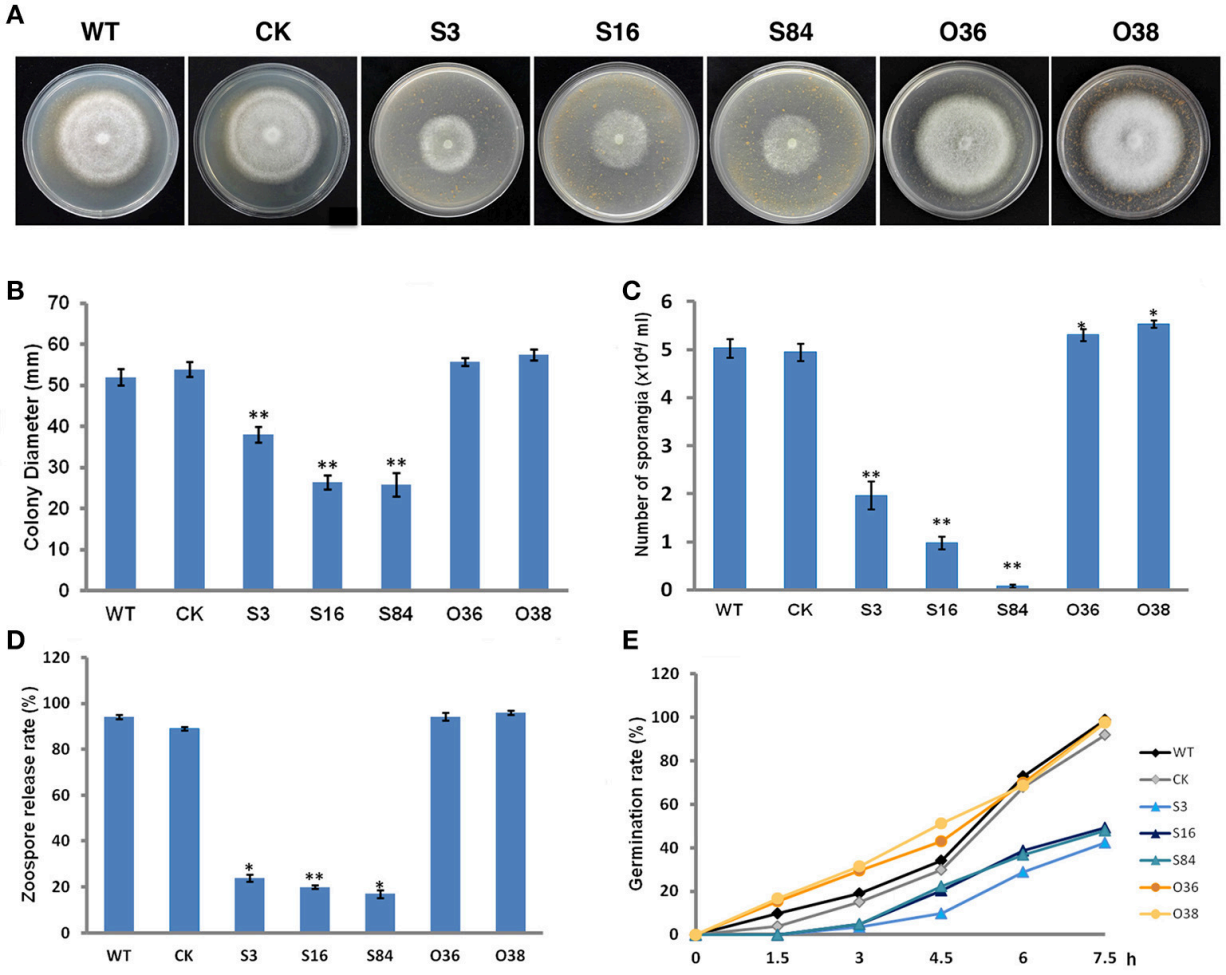
**Asexual phenotype characterization. (A)** Growth of the WT, CK, and the *PiLLP* transformants (S3, S16, S84, O36, and O38). Photos were taken at 7 days after incubation on 10% TRA medium. **(B)** Statistics of colony diameters was based on 7 days of growth on 10% TRA medium. **(C)** Sporangia of the WT, CK, and the *PiLLP* transformants (S3, S16, S84, O36, and O38) formed on 10 days old cultures were washed with water and then counted under the microscope. **(D)** Sporangia suspension was incubated in a plate for 2 h at 4°C. The ratios of the number of empty sporangia to the total number of sporangia were calculated for each strain. **(E)** Sporangia suspension was incubated in 200 g/L Peabroth at 18°C. The numbers of germinated and un-germinated sporangia were counted under a microscope at different time points (0, 1.5, 3, 4.5, 6, and 7.5 h), and the percentage of the germinated sporangia to the total number of sporangia were calculated for each strain. Three replicates were used for each treatment and the whole experiment was repeated once with three replicates. Bars represent the standard deviation of three replicates. Statistical significance was analyzed with Student's *t*-test between WT and each transformant (^*^P < 0.05, ^**^P < 0.01).

The colony diameters of STs (S3, S16, and S84) were significantly smaller than those of the WT and CK strains, whereas those of the OTs (O36 and O38) were not different from those of WT and CK, except that the mycelia of OTs colonies were denser (Figures 6A,B) (*P* < 0.01). In addition, the STs produced significantly less sporangia than the WT and CK strains (Figure 6C) (*P* < 0.01), and fewer zoospores were released from these sporangia in comparison with the WT and CK strains (Figure 6D). The OTs (O36 and O38) produced significantly more sporangia than WT and CK strains, and the number of zoospores released from these sporangia was almost equal to those of WT and CK strains (Figures 6C,D). In addition, the germination rates of the STs' sporangia were close to half the rates of WT and CK sporangia, whereas the germination rates of the OTs' sporangia were similar to those of the WT and CK strains (Figure 6E).

### Subcellular localization of PiLLP in *P. infestans*

**Figure 7:**
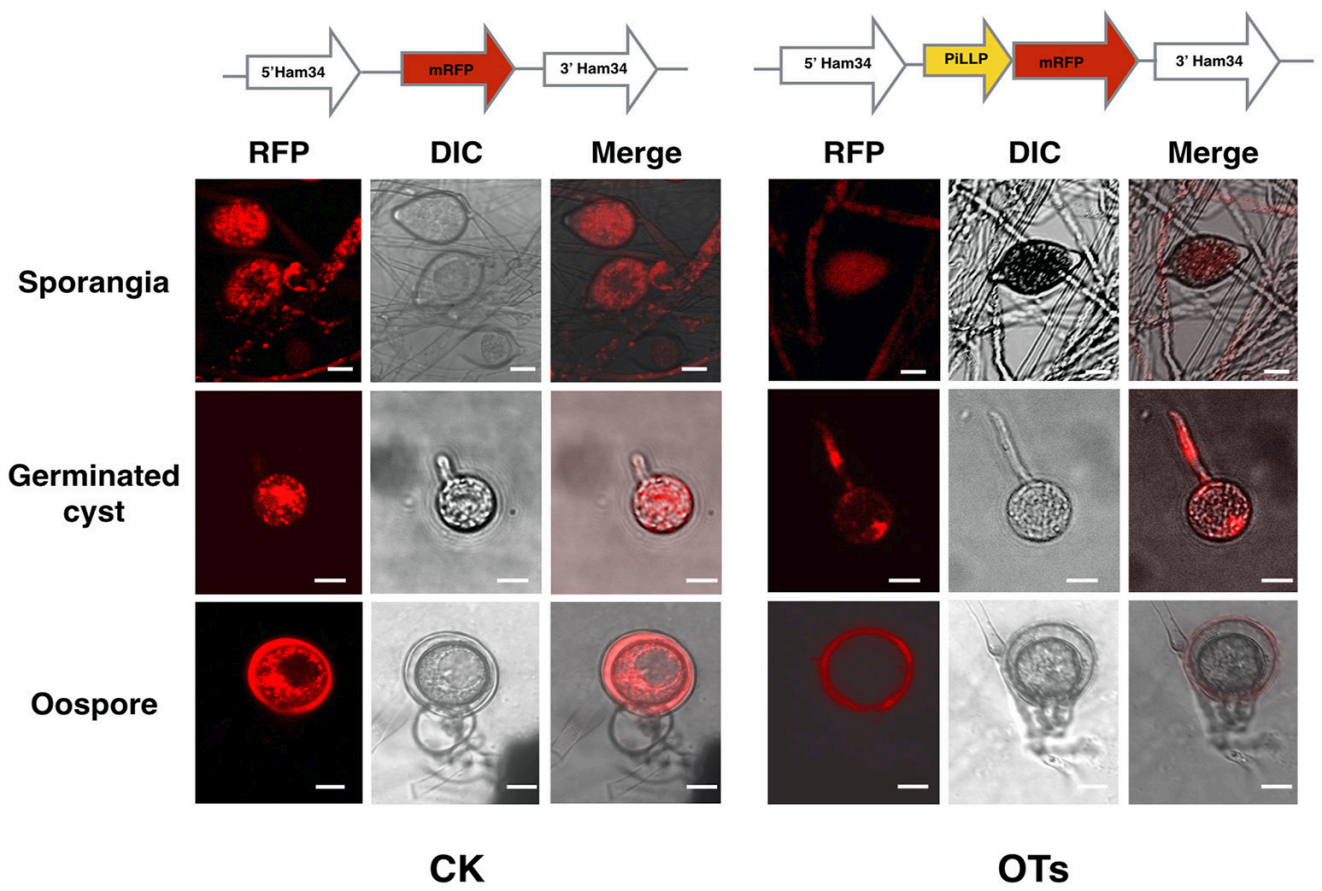
**Subcellular localization of PiLLP in oospores**. The red fluorescence was present in the cytoplasm of mycelia, sporangia, cysts and germinated cysts of control (CK) and the *PiLLP*-overexpressed transformants (OTs), but it was limited to the oogonium wall of OTs. The bars indicate 10 μm.

In order to investigate the subcellular localization of PiLLP in *P. infestans*, we labeled PiLLP by fusing *PiLLP* with the gene encoding monomeric red fluorescent protein (mRFP) to investigate the subcellular localization of the PiLLP protein in *P. infestans*. In asexual stages, the red fluorescence was observed in the cytoplasm of mycelia, sporangia, cysts, and germinated cysts. This fluorescence pattern was the same in the control strain CK and in three OT strains and therefore it is not possible to draw conclusions about the localization of PiLLP during asexual development. In the sexual stage, the fluorescence pattern in the three OT strains was different from the pattern seen in the CK strain. In the OT strains, the red fluorescence was specifically associated with the wall of the oogonia (Figure 7), indicating that PiLLP was localized in the oogonial walls.

### *PiLLP* participates in oxidative-stress resistance

**Figure 8:**
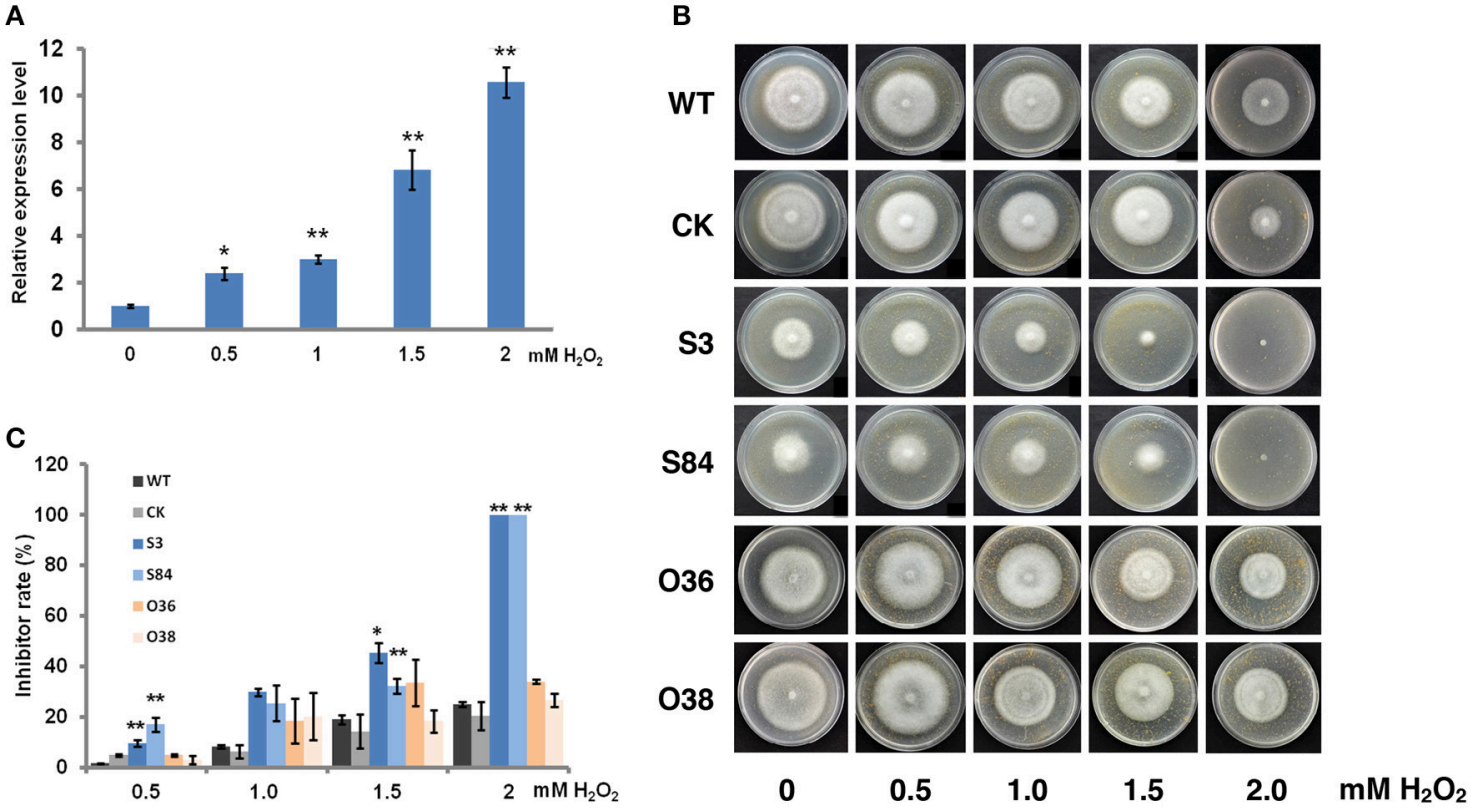
*****PiLLP*** participates in the response to oxidative stress. (A)** Levels of *PiLLP* expression in mycelia grown on medium amended with various concentration of hydrogen peroxide (H_2_O_2_). The qRT-PCR assay was performed using cDNAs synthesized from mycelial RNAs. The level of *PiLLP* expression was determined relative to that of *ef1*, and the relative gene expression levels shown were determined relative to expression on TRA medium alone (value, 1.0). **(B)** Colony expansion of WT, CK, and *PiLLP* transformants (S3, S84, O36, and O38) on TRA medium supplemented with 0, 0.5, 1.0, 1.5, and 2.0 mM H_2_O_2_. **(C)** Colony diameters were measured after 7 days of growth. Rates of growth inhibition were calculated for each isolates of each treatment relative to the growth on TRA medium. Three replicates were used for each treatment in these tests and the whole experiment was repeated once. Bars represent the standard deviation of three replicates. Statistical significance was analyzed using Student's *t*-test between WT and each transformant (^*^P < 0.05, ^**^P < 0.01).

To determine whether *PiLLP* is an oxidative-associated gene, the *PiLLP* expression levels in cultures treated with various concentration of H_2_O_2_ were investigated. The expression of *PiLLP* in the WT strain increased with increased H_2_O_2_ concentrations in the medium and was ~10-fold greater when the H_2_O_2_ concentration reached 2.0 mM (Figure 8A; Table S4). The growth rates of STs decreased greatly at 0.5, 1.0, 1.5, and 2.0 mM H_2_O_2_, and completely ceased at 2.0 mM H_2_O_2_. The inhibition rate of H_2_O_2_ on OTs growth was consistent with those of WT and CK strains (Figures 8B,C).

### *PiLLP* silencing reduces the virulence of *P. infestans*

**Figure 9:**
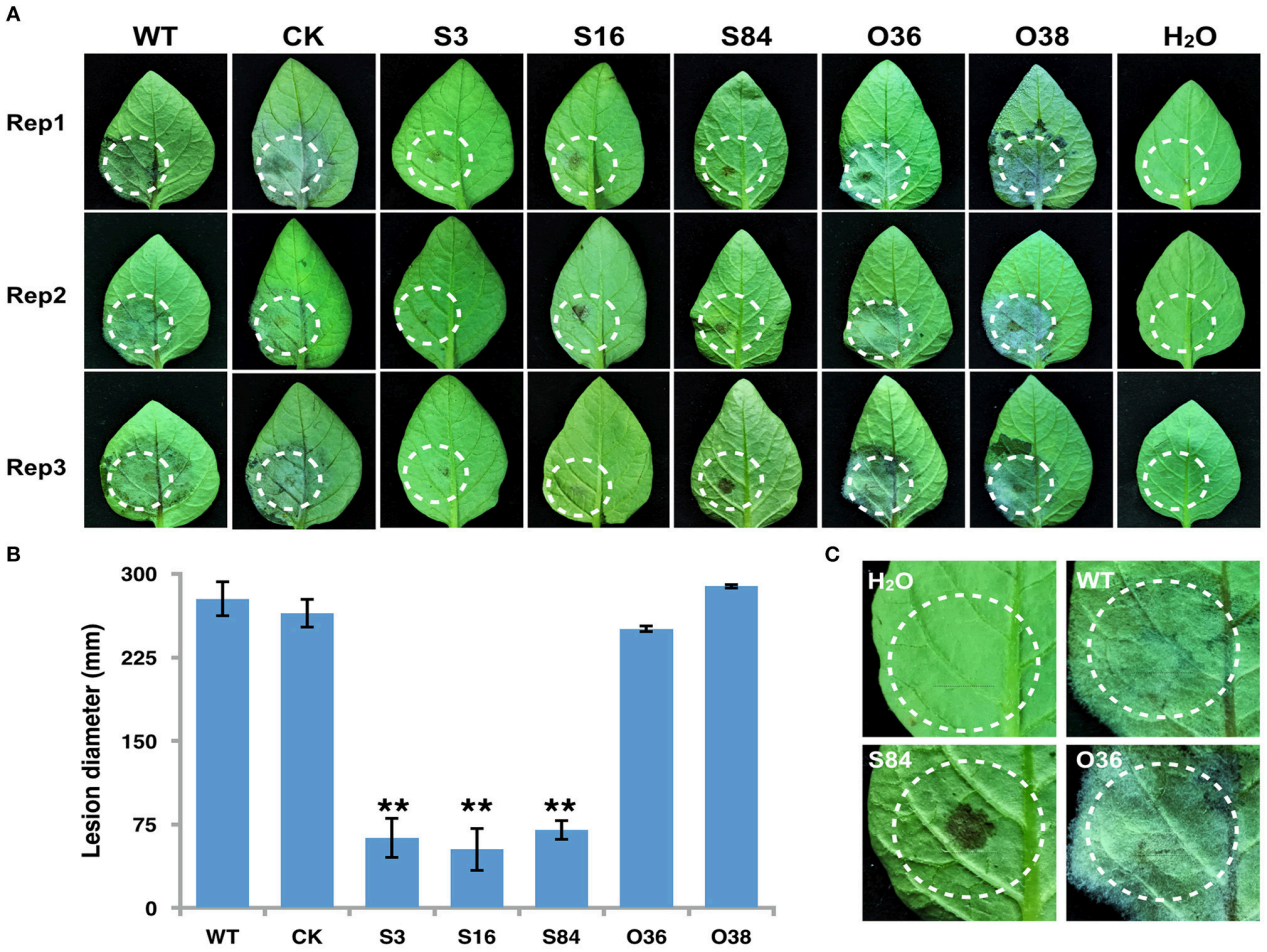
**Infection assay on detached potato leaves. (A)** Leaves of potato cultivar *Impala* were inoculated with sporangia (~100 in 10 μL for WT, CK, and *PiLLP*-overexpressed sporangia, and 200 for *PiLLP*-silenced transformant sporangia). Images were taken at 5 day after inoculation. **(B)** Sizes of lesions induced by different isolates. The diameter of each lesion was measured at 5 day after inoculation. Three replicates were used for each treatment in these tests and the whole experiment was repeated once. Bars represent the standard deviation of three replicates. Statistical significance was analyzed using Student's *t*-test (^**^P < 0.01). **(C)** The amplified image of inoculated leaves with different isolates (WT, S84, and O36).

**Figure 10:**
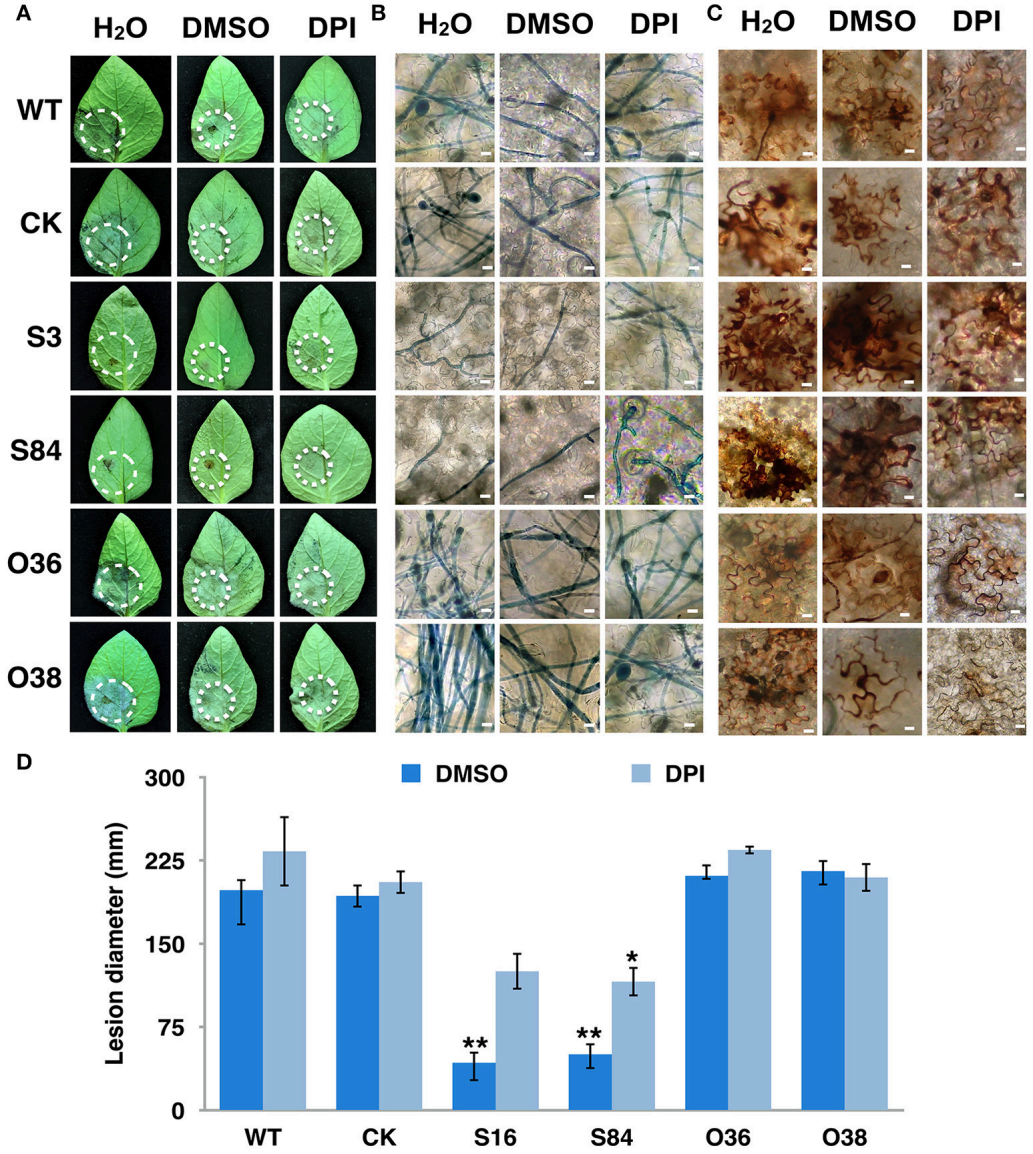
**Virulence of ***PiLLP***-silenced transformants restored by DPI treatment. (A)** Disease lesions induced by different isolates. Detached leaves of potato cultivar *Impala* were injected with H_2_O, 0.05% DMSO and 0.5 μM DPI. The treated leaves were inoculated on injection sites with sporangia [~100 in 10 μL for WT, CK, and *PiLLP*-overexpressed sporangia, whereas 200 for *PiLLP*-silenced transformant sporangia]. Pictures were taken 5 day after inoculation. **(B)** Microscopic observations of invasive hyphae in potato leave. The inoculated leaves were boiled for ~2 min in lactophenol-trypan blue and decolorized in 2.5 g/ml chloral hydrate for 30–40 min. **(C)** Detached leaves of *N. benthamiana* were injected with H_2_O, 0.05% DMSO and 0.5 μM DPI and then inoculated with sporangia of WT, CK, and *PiLLP* transformants. Twelve hours after inoculation, the leaves were incubated in 1 mg/mL DAB solution (pH 3.8), at room temperature for 8 h and destained with ethanol, and observed with a microscope. The bars indicate 10 μm. **(D)** Lesion diameter on DPI-untreated and -treated leaves induced by different isolates. The diameter of each lesion was measured at 5-day after inoculation. Three replicates were used for each treatment and the whole experiment was repeated once. Bars represent the standard deviation of three replicates. Statistical significance was analyzed using Student's *t*-test. The asterisk indicates the significant difference (^*^P < 0.05, ^**^P < 0.01). Two independent infection assays were performed with similar results.

To determine whether *PiLLP* is required for *P. infestans* pathogenicity, the detached leaves of potato (cv. Impala) were inoculated with equivalent amounts of germinated WT, CK, ST (S3, S16, and S84), and OT (O36 and O38) sporangia, independently. At 5 days post-inoculation, the typical disease symptoms of water-soaked lesions were observed on the leaves inoculated with WT, CK, and OTs (O36 and O38) strains, except that more aerial mycelia were observed on lesions induced by OTs. In contrast, only small brown spots were induced by the ST strains (Figures 9A–C). As the plant-derived ROS is an important defense mechanism of protecting plant from microorganism invasion, we pretreated the detached leaves with diphenyleneiodium (DPI), a flavoenzyme inhibitor that prevents the activation of NADPH oxidases, before inoculating with the STs. The lesion sizes on these treated leaves increased to half size of that induced by WT and were twice as large as those on the DPI-untreated leaves (Figures 10A,D). More mycelia were observed in cells at the inoculation site than in those of DPI-untreated leaves inoculated with STs (Figure 10B), and less reddish-brown precipitate was observed in the infection site of STs in DPI-treated leaves (Figure 10C). For leaves inoculated with OTs, no significant increase in lesions size was observed on DPI-treated leaves (Figure 10D). We observed the less dense of mycelia on lesions and in tissues at inoculation site treated with DPI or DMSO, and a lighter brown color in the infection site in DPI-treated leaves (Figures 10A–C). This finding indicates that the pathogenicity of STs could be partially restored by DPI treatment.

### PiLLP silencing reduces catalase genes expression level and catalase activities

**Figure 11:**
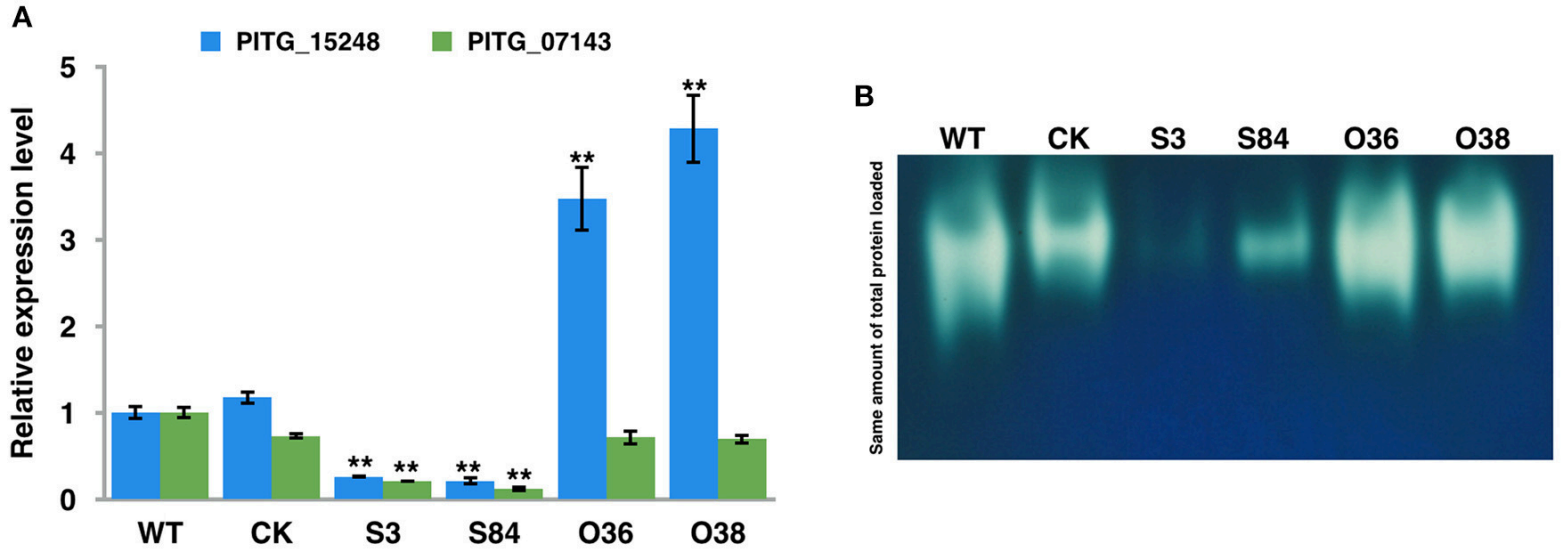
*****PiLLP-***silenced transformants with reduced catalase genes expression level and lower catalase activities. (A)** The expression levels of two catalase genes (*PITG_15248 and PITG_07143*) in WT, CK, and transformants (S3, S84, O36, and O38). The qRT-PCR assay was performed using cDNAs synthesized from mycelial RNAs of different isolates (WT, CK, STs and OTs). The levels of *PITG_15248 or PITG_07143* were determined relative to that of *ef1*, and the expression level of WT was used as a reference (value, 1.0). **(B)** In-gel assay of catalase activity. Equal amounts of total protein (20 μg) of different isolates (WT, CK, STs, and OTs) were loaded into Native-PAGE. The gel was immersed in 7 mM H_2_O_2_ for 10 min after electrophoresis, and then incubated in a 1/1 mixture of freshly prepared 1% potassium hexacyanoferrate (III) and 1% iron (III) chloride hexahydrate. Blue color was observed in the gel except at zones where H_2_O_2_ was decomposed by catalases. Three replicates were used for each treatment in these tests and the whole experiment was repeated with a different set of biological samples. Bars represent the standard deviation of three replicates. Statistical significance was analyzed using Student's *t*-test between WT and each transformant (^**^P < 0.01).

As the transcriptome analysis of *P. infestans* showed the up-regulation of two catalase genes, (*PITG_15248* and *PITG_07143*) during the early infection stages (Haas et al., 2009), we then analyzed the expression level of these genes in WT, CK, and transformants. The expression levels of these two genes were significantly decreased in STs, whereas the expression level of *PITG_15248* was significantly increased in OTs, compared with those in WT and CK (Figure 11A; Table S4). We then further investigated the activity of catalases of WT, CK, and transformants by analyzing the total catalase activities of the crude proteins extracts from these isolates with PAGE under non-denatured conditions (Figure 11B). As shown in Figure 11B, the bands of WT and CK strains were thicker and brighter than those of STs, but significantly lighter and thinner than those of OTs, indicating the decrease of catalase activity in STs and the increase of catalase activity in OTs.

## Discussion

### *LLP* is required in the reproduction of oomycetes

In this study, we identified a *P. infestans* ortholog of the plant *LLP* gene. Bioinformatics analysis showed that LLP is highly conserved in oomycetes and contains mixed GGX and GX repeats. The protein is phylogenetically related to the LLP, Class V GRPs in plants, which function is not clear. The similarity between PiLLP and the rest of LLPs ranged from 32.1 to 90.9%. In animals and humans, loricrin is a highly insoluble structural protein and is the main component of the cornified cell envelope, which functions as structural reinforcement (Kalinin et al., 2001). Together with the indication of its localization in oogonial walls, this suggests that LLP is a structural component in the ooginial wall. The increase in *PiLLP* expression in asexual and sexual development stages also indicates that *PiLLP* is required for reproduction of *P. infestans*. Thus, the decrease in the colony expansion rate, the formation rates of sporangia and oospores, and the zoospore-releasing rate in *PiLLP*-silenced transformants is not surprising. However, further investigation is needed to confirm its localization on oogonia wall.

### *PiLLP* is involved in oospore formation

Previous studies showed that various types of proteins constitute 12.5% of the oospore–oogonium wall, and the most abundant amino acid residue in these proteins is glycine followed by alanine, arginine, aspartic acid, and glutamic acid (Lippman et al., 1974), which is consistent with the amino acid composition of PiLLP. The presence of layered patches of electron-dense deposits at the inner layer of oogonial wall likely belong to osmiophilic proteins or lipids, but these layered patches of electron-dense deposits are absent in the walls of hyphae, sporangia, zoospores, and cysts (Hegnauer and Hohl, 1978).

Here, some STs could form many normal immature oogonia but ~45% of these oogonia could not form oospores. The simultaneous loss of lipid bodies and turgor in the oogonia indicates that the loss of cytoplasm content and/or turgor is related to defective oospore formation in the STs. Previous studies on the gametangial development in *Phytophthora* spp. showed that fertilization occurs before oosphere formation, and the antheridial nucleus enters oogonia through the fertilization tube, rupturing the oogonial wall (Figure 5D; Hemmes and Bartnicki-Garcia, 1975; Hemmes and Ribeiro, 1977). As a consequence, the ruptured area on the oogonial wall requires patching to avoid leaking cytoplasm and nuclei from oogonium. However, the shortage of structural proteins in the oogonial wall may result in an unpatched oogonial wall break, ultimately leading to the failure of oospore formation. The Nile red staining of developing sexual apparatus provided evidence supporting this speculation. As shown, the red fluorescence-emitting substance indicated that the lipid bodies were present only in antheridia attached to the 17-day-old (late stage) partially emptied oogonia from STs, not in those attached to the oogonia with mature oospores, indicating the translocation of lipid bodies from the later stage oogonium to the antheridium (Figure 5E).

Thus, this study suggested that PiLLP is a structural component of the oogonial wall and is likely related to the electron-dense deposits specific to the oogonial wall. However, further investigations are needed to illustrate the components of the electron-dense deposits on the inner oogonial wall. Previously, a small tyrosine rich protein, PoStr, was shown to be involved in the oogonial and oospore wall formation in *Phythium* sp. (Grenville-Briggs et al., 2013).

### *PiLLP* is involved in scavenging plant ROS

The accumulation of plant-derived ROS at the infection site is an early immunity response in plants (Mellersh et al., 2002; Torres and Dangl, 2005). To successfully invade plant cells, plant pathogens must avoid damage from ROS through self-protection or scavenging ROS, or both. In plant fungal pathogens, several genes, such as *Yap1* in *Ustilago maydis* (Molina and Kahmann, 2007) and *DES1* in *Magnaporthe oryzae* (Chi et al., 2009) are responsible for ROS detoxification at the infection site and are required for virulence.

STs being more sensitive to H_2_O_2_ than the WT and OTs strains, and the induced expression of *PiLLP* in the WT strain being highly increased as the H_2_O_2_ concentration increased in the medium, indicated that *PiLLP* is involved in resistance to H_2_O_2._ Additionally, a greater amount of H_2_O_2_ accumulation and a decline in mycelial expansion occurred in infection site tissues inoculated with STs than in those inoculated with WT, CK, and OTs. We also showed that the reduced pathogenicity of the STs could be partially restored with DPI treatments. These results indicate that the expansion of STs in infection site tissues is limited partially because of the attack of host-derived ROS. Therefore, we speculate that *PiLLP* is involved in scavenging plant ROS during infection. A transcriptome analysis of *P. infestans* revealed that two catalase genes (*PITG_15248* and *PITG_07143*) were up-regulated during early infection stages (Haas et al., 2009). Here, we found that the expression levels of these two genes were decreased in STs compared with those in WT, CK, and OTs. We further investigated the activity of catalase in different isolates and found the decrease of catalase activity in STs and the increase of catalase activity in OTs compared with WT and CK. These results suggested that *PiLLP* affected the pathogenicity of isolates through affecting the catalase activity, which is important for scavenging plant ROS.

In the pathogenicity test, we placed a double amount of STs sporangia on the leaves to compensate for the lower germination rate of STs sporangia, which was around 50% of the WT and OTs. The growth rates of the two STs used in the pathogenicity test were about 50% lower than those of WT and OTs. Thus the defect in growth rate of STs may explain why pathogenicity of STs was not fully restored by DPI treatment. In *P. infestans*, two homologous genes of *PiLLP* were detected, and the expression of two genes with high sequence identities to *PiLLP* (*PITG_10076*, 51.1%; *PITG_00234*, 46.8%) in WT and STs did not show significant difference (Table S5).

Sequence analysis revealed that PiLLP does not contain a signal peptide or any structural domain with known functions but does contain a number of mixed GGX/GXGX repeats. Therefore, it is less likely that PiLLP could function as a secreted effector inducing plant cell death or as a cell wall-degrading enzyme. So, it is reasonable that the overexpression of this gene didn't increase the virulence of the OTs. DPI is a flavoenzyme inhibitor that can scavenge the host-derived ROS. As the pathogenicity test showed that WT, CK, and OTs can scavenge the host-derived ROS by itself, but not STs, it is expected that DPI treatment experiments have an obvious effect only on STs, but not on OTs.

Previous work suggested that the heat shock transcription factor PsHSF1 and histone acetyltransferase PsGcn5 were involved in oxidative stress responses and detoxifying oxidative bursts (Sheng et al., 2015; Zhao et al., 2015). Our study showed that *PiLLP* is involved in the H_2_O_2_ resistance and ROS scavenging, which are likely caused by affecting the transcription level of catalase genes. Previous research has shown the diverse functions of the GRPs in plants, but the function of the glycine-rich repeats in these proteins is not known (Mangeon et al., 2010). One speculation is that the glycine-rich repeats are necessary for interaction with other proteins in a multicomponent complex (Mangeon et al., 2010). However, further studies are expected to resolve the detailed mechanisms involved.

## Author contributions

Conceived and designed the study: LG Performed the experiment: TG Participate in some experiments: XW and KS. Edited and contributed to the manuscript: TG, XW, WS, and LG. All authors read and approved the final manuscript.

## Funding

This research was funded in part by the National Natural Science Foundation of China (30270862) and the Chinese Universities Scientific Fund (2015NX005).

### Conflict of interest statement

The authors declare that the research was conducted in the absence of any commercial or financial relationships that could be construed as a potential conflict of interest.

## Supplementary material

Table S1 **The LLP genes of ***P. infestans*** and other species**.

Table S2 **Primers used for all experiments**.

Table S3 **Percentage of aborted oospores of WT, CK, and transformants**.

Table S4 **Gene expression data of qRT-PCR in this study**.

Table S5 **Expression level of ***PITG_10076*** and ***PITG_00234*****.

## References

[B1] Adl S. M. Simpson A. G. Farmer M. A. Andersen R. A. Anderson O. R. Barta J. R.. (2005). The new higher level classification of eukaryotes with emphasis on the taxonomy of protists. J. Eukaryot. Microbiol. 52, 399–451. 10.1111/j.1550-7408.2005.00053.x 16248873

[B2] Apel K. Hirt H. (2004). Reactive oxygen species: metabolism, oxidative stress, and signal transduction. Annu. Rev. Plant Biol. 55, 373–399. 10.1146/annurev.arplant.55.031903.141701 15377225

[B3] Apostol I. Heinstein P. F. Low P. S. (1989). Rapid stimulation of an oxidative burst during elicitation of cultured plant cells: role in defense and signal transduction. Plant Physiol. 90, 109–116. 10.1104/pp.90.1.109 16666719 PMC1061684

[B4] Aylor D. E. Fry W. E. Mayton H. Andrade-Piedra J. (2001). Quantifying the rate of release and escape of *Phytophthora infestans* sporangia from a potato canopy. Phytopathology 91, 1189–1196. 10.1094/PHYTO.2001.91.12.1189 18943334

[B5] Baldauf S. L. Roger A. J. Wenk-Siefert I. Doolittle W. F. (2000). A kingdom-level phylogeny of eukaryotes based on combined protein data. Science 5493, 972–977. 10.1126/science.290.5493.972 11062127

[B6] Bocca S. N. Magioli C. Mangeon A. Junqueira R. M. Cardeal V. Margis R. (2005). Survey of glycine-rich proteins (GRPs) in the *Eucalyptus* expressed sequence tag database (ForEST). Genet. Mol. Biol. 28, 608–624. 10.1590/S1415-47572005000400016

[B7] Bourke A. (1993). The visitation of God? The potato and the Great Irish Famine. Dublin: The Lillput Press.

[B8] Brouwer H. Coutinho P. M. Henrissat B. de Vries R. P. (2014). Carbohydrate-related enzymes of important *Phytophthora* plant Pathogens. Fungal Genet. Bio. 72, 192–200. 10.1016/j.fgb.2014.08.011 25192612

[B9] Cassab G. I (1998). Plant cell wall proteins. Annu. Rev. Plant Physiol. Plant Mol. Biol. 49, 281–309. 10.1146/annurev.arplant.49.1.281 15012236

[B10] Chi M. H. Park S. Y. Kim S. Lee Y. H. (2009). A Novel pathogenicity gene is required in the rice blast fungus to suppress the basal defenses of the host. PLoS Pathog. 5:e1000401. 10.1371/journal.ppat.1000401 19390617 PMC2668191

[B11] Cross A. R. Jones O. T. G. (1986). The effect of the inhibitor diphenylene iodonium on the superoxide-generating system of neutrophils. Biochem. J. 237, 111–116. 10.1042/bj2370111 3800872 PMC1146954

[B12] de Oliveira D. E. Franco L. O. Simoens C. Seurinck J. Coppitiers J. Botterman J.. (1993). Inflorescence-specific genes from *Arabidopsis thaliana* encoding glycine-rich proteins. Plant J. 3, 495–507. 10.1046/j.1365-313X.1993.03040495.x 8220457

[B13] Doke N. Miura Y. Sanchez L. Park H. Noritake T. Yoshioka H. (1996). The oxidative burst protects plants against pathogen attack: mechanism and role as an emergency signal for plant biodefence-a review. Gene 179, 45–51. 10.1016/S0378-1119(96)00423-4 8955628

[B14] Dong S. Yin W. Kong G. Yang X. Qutob D. Chen Q.. (2011). *Phytophthora sojae* avirulence effector Avr3b is a secreted NADH and ADP-ribose pyrophosphorylase that modulates plant immunity. PLoS Pathog. 7:e1002353. 10.1371/journal.ppat.1002353 22102810 PMC3213090

[B15] Eloy Y. R. Vasconcelos I. M. Barreto A. L. Freire-Filho F. R. Oliveira J. T. (2015). H_2_O_2_ plays an important role in the lifestyle of *Colletotrichum gloeosporioides* during interaction with cowpea [*Vigna unguiculata* (L.) Walp.]. Fungal Biol. 119, 747–757. 10.1016/j.funbio.2015.05.001 26228563

[B16] Fabritius A. L. Cvitanich C. Judelson H. S. (2002). Stage-specific gene expression during sexual development in *Phytophthora infestans*. Mol. Microbiol. 45, 1057–1066. 10.1046/j.1365-2958.2002.03073.x 12180924

[B17] Gamboa-Meléndez H. Huerta A. I Judelson H. S. (2013). bZIP transcription factors in the oomycete *Phytophthora infestans* with novel DNA-binding domains are involved in defense against oxidative stress. Eukaryotic Cell 12, 1403–1412. 10.1128/EC.00141-13 23975888 PMC3811335

[B18] Gao J. Cao M. Ye W. Li H. Kong L. Zheng X.. (2015). PsMPK7, a stress-associated mitogen-activated protein kinase (MAPK) in *Phytophthora sojae*, is required for stress tolerance, reactive oxygenated species detoxification, cyst germination, sexual reproduction and infection of soybean. Mol. Plant Pathol. 16, 61–70. 10.1111/mpp.12163 24889742 PMC6638454

[B19] Gavino P. D. Smart C. D. Sandrock R. W. Miller J. S. Hamm P. B. Lee T. Y. (2000). Implications of sexual reproduction for *Phytophthora infestans* in the United States: generation of an aggressive lineage. Plant Dis. 84, 731–735. 10.1094/PDIS.2000.84.7.731 30832099

[B20] Goddemeier M. L. Wulff D. Feix G. (1998). Root-specific expression of a *Zea mays* gene encoding a novel glycine-rich protein, zmGRP3. Plant Mol. Biol. 36, 799–802. 10.1023/A:1005998804622 9526513

[B21] Grenville-Briggs L. J. Horner N. R. Phillips A. J. Beakes G. W. West P. V (2013). A family of small tyrosine rich proteins is essential for oogonial and oospore wall development of the mycoparasitic oomycete *Pythium Oligandrum*. Fungal Biol. 117, 163–172. 10.1016/j.funbio.2013.01.001 23537873

[B22] Guo L. Y. Zhu X. Q. Hu C. H. Ristaino J. B. (2010). Genetic structure of *Phytophthora infestans* populations in China indicates multiple migration events. Phytopathology 100, 997–1006. 10.1094/PHYTO-05-09-0126 20839935

[B23] Haas B. J. Kamoun S. Zody M. C. Jiang R. H. Handsaker R. E. Cano L. M.. (2009). Genome sequence and analysis of the Irish potato famine pathogen Phytophthora infestans. Nature 461, 393–398. 10.1038/nature08358 19741609

[B24] Han M. Liu G. Li J. P. Govers F. Zhu X. Q. Shen C. Y. (2012). *Phytophthora infestans* field isolates from Gansu Province, China are Genetically highly diverse and show a high frequency of self fertility. *J*. Eukaryot. Microbiol. 60, 79–88. 10.1111/jeu.12010 23194320

[B25] Harris H. Hopkinson D. A. (1987). Chapter 4 Handbook of Enzyme Electrophoresis in Human Genetics,. Amsterdam: North-Holland Publishing Co.

[B26] Hegnauer H. Hohl H. R. (1978). Cell wall architecture of sporangia, chlamydospores, oogonia and oospores in *Phytophthora*. Exp. Mycol. 2, 216–233. 10.1016/S0147-5975(78)80015-2

[B27] Hemmes D. E. (1983). Cytology of phytophthora, in Phytophthora: Its Biology, Taxonomy, Ecology, and Pathology, eds Erwin D. C. Bartnicki-Garcia S. Tsao P. H. (St. Paul, MN: The American Phytopathological Society), 28–34.

[B28] Hemmes D. E. Bartnicki-Garcia S. (1975). Electron microscopy of gametangial interaction and oospore development in *Phytophthora capsici*. Arch. Microbiol. 103, 91–112. 10.1007/BF00436336

[B29] Hemmes D. E. Ribeiro O. K. (1977). Electron microscopy of early gametangial interaction in Phytophthora megasperma var. sojae. Can. J. Bot. 55, 436–447. 10.1094/PDIS.2000.84.11.1190

[B30] Ho H. H. (1979). Electron microscope studies of oogonial wall and elemental composition of oogonia in *phytophthora*. Mycopathologia 68, 17–21. 10.1007/BF00490386

[B31] Hohl D. Olano B. R. de Viragh P. A. Huber M. Detrisac C. J. Schnyder U. W.. (1993). Expression patterns of loricrin in various species and tissues. Differentiation 54, 25–34. 10.1111/j.1432-0436.1993.tb00656.x 8405772

[B32] Kalinin A. Marekov L. N. Steinert P. M. (2001). Assembly of the epidermal cornified cell envelope. *J*. Cell. Sci. 114, 3069–3070. 10.1242/jcs.114.17.3069 11590230

[B33] Karling J. S. (1981). Predominantly holocarpic and eucarpic simple biflagellate phycomycetes. J. Carmer. 252, 557–558.

[B34] Koch E. Slusarenko A. (1990). Arabidopsis is susceptible to infection by a downy mildew fungus. Plant Cell 2, 437–445. 10.1105/tpc.2.5.437 2152169 PMC159900

[B35] Küster H. Schröder G. Frühling M. Pich U. Rieping M. Schubert I.. (1995). The nodule- specific *VfENOD-GRP3* gene encoding a glycine-rich early nodulin is located on chromosome I of *Vicia faba* L. and is predominantly expressed in the interzone II-III of root nodules. Plant Mol. Biol. 28, 405–421. 10.1007/BF00020390 7632912

[B36] Larkin M. A. Blackshields G. Brown N. P. Chenna R. McGettigan P. A. McWilliam H.. (2007). Clustal W and Clustal X version 2.0. Bioinformatics 23, 2947–2948. 10.1093/bioinformatics/btm404 17846036

[B37] Lippman E. Erwin D. C. Bartnicki-Garcia S. (1974). Isolation and chemical composition of oospore-oogonium walls of *Phytophthora magasperma* var. sojae. J. Gen. Microbiol. 80, 131–141. 10.1099/00221287-80-1-131

[B38] Liu Z. Friesen T. (2012). DAB staining and visualization of hydrogen peroxide in wheat leaves. Bioprotocol 2:e309 10.21769/bioprotoc.309

[B39] Lledıas F. Rangel P. Hansberg W. (1998). Oxidation of Catalase by Singlet Oxygen. J. Biol. Chem. 273, 10630–10637. 10.1074/jbc.273.17.10630 9553125

[B40] Mangeon A. Junqueira R. M. Sachetto-Martins G. (2010). Functional diversity of the plant glycine-rich proteins superfamily. Plant Signal. Behav. 5, 99–104. 10.4161/psb.5.2.10336 20009520 PMC2884108

[B41] Mangeon A. Pardal R. Menezes-Salgueiro A. D. Duarte G. L. de Seixas R. Cruz F. P.. (2016). *AtGRP3* Is implicated in root size and aluminum response pathways in *Arabidopsis*. PLoS ONE 11:e0150583. 10.1371/journal.pone.0150583 26939065 PMC4777284

[B42] Mayfield J. A. Preuss D. (2000). Rapid initiation of *Arabidopsis* pollination requires the oleosin-domain protein GRP17. Nat. Cell. Biol. 2, 128–130. 10.1038/35000084 10655594

[B43] Mayton H. Smart C. D. Moravec B. C. Mizubuti E. S. G. Muldoon A. E. Fry W. E. (2000). Oospore survival and pathogenicity of single oospore recombinant progeny from a cross involving the US-8 and US-17 lineages of *Phytophthora infestans*. Plant Dis. 84, 1190–1196. 10.1094/PDIS.2000.84.11.1190 30832166

[B44] Mcleod A. Fry B. A. Zuluaga A. P. Kevin M. Fry W. E. (2008). Toward improvements of oomycete transformation protocols. J. Eukaryot. Microbiol. 55, 103–109. 10.1111/j.1550-7408.2008.00304.x 18318863

[B45] Mellersh D. G. Foulds I. V Higgins V. J. Heath M. C. (2002). H_2_O_2_ plays different roles in determining penetration failure in three diverse plant-fungal interactions. Plant J. 29, 257–268. 10.1046/j.0960-7412.2001.01215.x 11844104

[B46] Molina L. Kahmann R. (2007). An *Ustilago maydis* gene involved in H_2_O_2_ detoxification is required for virulence. Plant Cell 19, 2293–2309. 10.1105/tpc.107.052332 17616735 PMC1955693

[B47] Nithya S. Radhika T. Jeddy N. (2015). Loricrin – an overview. J. Oral. Maxillofac. Pathol. 19, 64–68. 10.4103/0973-029X.157204 26097310 PMC4451671

[B48] Park S. J. Kwak K. J. Oh T. R. Kim Y. O. Kang H. (2009). Cold shock domain proteins affect seed germination and growth of *Arabidopsis thaliana* under abiotic stress conditions. Plant Cell Physiol. 50, 869–878. 10.1093/pcp/pcp037 19258348

[B49] Prakob W. Judelson H. S. (2007). Gene expression during oosporogenesis in heterothallic and homothallic *Phytophthora*. Fungal Genet. Biol. 44, 726–739. 10.1016/j.fgb.2006.11.011 17215149

[B50] Ribeiro O. K. (1978). A Source Book of the Genus Phytophthora. Vaduz: Cramer.

[B51] Ribeiro O. K. (1983). Physiology of asexual sporulation and spore germination in *Phytophthora*, in Phytophthora: Its Biology, Taxonomy, Ecology, and Pathology, eds Erwin D. C. Bartnicki-Garcia S. Tsao P. H. (St. Paul, MN: The American Phytopathological Society), 58–60.

[B52] Ringli C. Hauf G. Keller B. (2001). Hydrophobic interactions of the structural protein GRP1.8 in the cell wall of protoxylem elements. Plant Physiol. 125, 673–682. 10.1104/pp.125.2.673 11161025 PMC64869

[B53] Ryser U. Keller B. (1992). Ultrastructural localization of a bean glycine-rich protein in unlignified primary walls of protoxylem cells. Plant Cell. 4, 773–783. 10.1105/tpc.4.7.773 12297662 PMC160173

[B54] Ryser U. Schorderet M. Zhao G. F. Studer D. Ruel K. Hauf G.. (1997). Structural cell-wall proteins in protoxylem development: evidence for a repair process mediated by a glycine-rich protein. Plant J. 12, 97–111. 10.1046/j.1365-313X.1997.12010097.x 9263454

[B55] Sanju S. Siddappa S. Thakur A. Shukla P. K. Srivastava N. Pattanayak D.. (2015). Host-mediated gene silencing of a single effector gene from the potato pathogen *Phytophthora infestans* imparts partial resistance to late blight disease. Funct. Integr. Genomics. 15, 697–706. 10.1007/s10142-015-0446-z 26077032

[B56] Sheng Y. Wang Y. Meijer H. J. Yang X. Hua C. Ye W.. (2015). The heat shock transcription factor PsHSF1 of *Phytophthora sojae* is required for oxidative stress tolerance and detoxifying the plant oxidative burst. Environ. Microbiol. 17, 1351–1364. 10.1111/1462-2920.12609 25156425

[B57] Sogin M. L. Silberman J. D. (1998). Evolution of the protists and protistan parasites from the perspective of molecular systematics. Int. J. Parasitol. 28, 11–20. 10.1016/S0020-7519(97)00181-1 9504331

[B58] Sparrow F. K. (1960). Aquatic Phycomycetes 2nd Revised Edition. Ann Arbor, MI: University of Michigan Press, 1187.

[B59] Tamura K. Peterson D. Peterson N. Stecher G. Nei M. Kumar S. (2011). MEGA5: molecular evolutionary genetics analysis using maximum likelihood, evolutionary distance, and maximum parsimony methods. Mol. Biol. Evol. 28, 2731–2739. 10.1093/molbev/msr121 21546353 PMC3203626

[B60] Torres M. A. Dangl J. L. (2005). Functions of the respiratory burst oxidase in biotic interactions, abiotic stress and development. Curr. Opin. Plant Biol. 8, 397–403. 10.1016/j.pbi.2005.05.014 15939662

[B61] Torto T. A. Rauser L. Kamoun S. (2002). The pipg1 gene of the oomycete *Phytophthora infestans* encodes a fungal-like endopolygalacturonase. Curr. Genet. 40, 385–390. 10.1007/s00294-002-0272-4 11919677

[B62] Turkensteen L. J. Flier W. G. Wanningen R. Mulder A. (2000). Production, survival and infectivity of oospores of *Phytophthora infestans*. Plant Pathol. 49, 688–696. 10.1046/j.1365-3059.2000.00515.x

[B63] Ueki S. Citovsky V. (2002). The systemic movement of a tobamovirus is inhibited by a cadmium-ion-induced glycine-rich protein. Nat. Cell Biol. 4, 478–486. 10.1038/ncb806 12055637

[B64] Wang Y. Dong Q. Ding Z. Gai K. Han X. Kaleri F. N.. (2016). Regulation of *Neurospora* Catalase-3 by global heterochromatin formation and its proximal heterochromatin region. Free Radic Biol. Med. 99, 139–152. 10.1016/j.freeradbiomed.2016.07.019 27458122

[B65] Yoon H. S. Hackett J. D. Pinto G. Bhattacharya D. (2002). The single, ancient origin of chromist plastids. Proc. Natl. Acad. Sci. U.S.A. 99, 15507–15512. 10.1073/pnas.242379899 12438651 PMC137747

[B66] Zhao W. Dong S. Ye W. Hua C. Meijer H. J. Dou X.. (2011). Genome-wide identification of *Phytophthora sojae* SNARE genes and functional characterization of the conserved SNARE PsYKT6. Fungal Genet. Biol. 48, 241–251. 10.1016/j.fgb.2010.11.006 21109013

[B67] Zhao W. Wang T. Liu S. S. Chen Q. Q. Qi R. D. (2015). The histone acetyltransferase PsGcn5 mediates oxidative stress response and is required for full virulence of *Phytophthora sojae*. Microb. Pathog. 87, 51–58. 10.1016/j.micpath.2015.07.015 26209751

