# Supplementary material for: The Loricrin-Like Protein (LLP) of Phytophthora infestans Is Required for Oospore Formation and Plant Infection
Source: Front Plant Sci. 2017 Feb 9;8:142. doi: 10.3389/fpls.2017.00142 (PMC5298957; doi:10.3389/fpls.2017.00142)
Supplement: Table S1 — The LLP genes of P. infestans and other species. [file Table1.DOCX]

| Organism category | | Organism species |  | Gene ID |
| --- | --- | --- | --- | --- |
|  | Oomycete | *Phytophthora infestans* |  | PITG_15862 |
|  |  | *Phytophthora parasitica* |  | PPTG_12643 |
|  |  | *Phytophthora ramorum* |  | Phyra74197 |
|  |  | *Hyaloperonospora parasitica* |  | HpaG811980 |
|  |  | *Phytophthora sojae* |  | Physo3_558230 |
|  |  | *Phytophthora cinnamomi* |  | e_gw1.108.238.1 |
|  |  | *Pythium ultimum* |  | PYU1_G001136 |
|  |  | *Alugo laibachii* |  | AlNc14C22G2223 |
|  |  | *Saprolegnia parasitica* |  | SPRG_11404 |
|  | Plant | *Sorhum bicolor* |  | Sb10g020110 |
|  |  | *Arabidopsis thaliana* |  | AT1G64140 |
|  |  | *Zea mays* |  | Zm.30957 |
|  |  | *Oryza sativa* |  | 4329752 |
|  |  | *Arabidopsis thaliana* |  | AT5G09670 |
|  |  | *Arabidopsis thaliana* |  | AT5G64550 |
|  |  | *Arabidopsis thaliana* |  | AT2G05520 |
|  |  | *Arabidopsis thaliana* |  | AT3G20470 |
|  |  | *Arabidopsis thaliana* |  | AT5G07530 |
|  |  | *Oryza sativa* |  | 4352868 |
|  |  | *Arabidopsis thaliana* |  | AT4G13850 |
|  |  | *Arabidopsis thaliana* |  | AT4G39260 |
|  | Diatom | *Thalassiosira pseudonana* |  | THAPSDRAFT 268581 |
|  | Protist | *Tetrahymena thermophila* |  | TTHERM 00532690 |
|  |  | *Paramecium tetraurelia* |  | GSPATT0018122001 |

**Table S1** The LLP genes of *P. infestans* and other species.
