# Supplementary material for: The Loricrin-Like Protein (LLP) of Phytophthora infestans Is Required for Oospore Formation and Plant Infection
Source: Front Plant Sci. 2017 Feb 9;8:142. doi: 10.3389/fpls.2017.00142 (PMC5298957; doi:10.3389/fpls.2017.00142)
Supplement: Table S2 — Primers used for all experiments. [file Table2.DOCX]

**Table S2** Primers used for all experiments

| Primers | Forward Primer (5'→3') | Reverse Primer (5'→3') | Purpose |
| --- | --- | --- | --- |
| PiLLP-5’F/5’R | TAACCGCGGTCCTCCTCTCTGCGT | TGTACTACTGCTAAGGTGGGGCTGTAA | 5'UTR region of *PiLLP* cloning |
| PiLLP-3’F/3’R | AGCAGTAGTACAGCAACGAGGCTTCGA | ACTCGTACGACGACACTTGAAACATCG | 3'UTR region of *PiLLP* cloning |
| PiLLP-LF /LR | CGCATCGATATGACTGTCCGCACGCA | ATACGTACGGAAGCACCGCGCCGCC | ORF region of *PiLLP* cloning |
| PiLLP-qF/qR | CAGTCTCCCGTCACCAGTCC | GGCAGTCGGCAATACCACAT | RT-PCR of *PiLLP* |
| TOR-F/R | GACCTTCACTCTCACCGACAA | TCCTTGATGACGTCCTCGGA | Primers for verifying *PiLLP* localization |
| TOR-F/SR | GACCTTCACTCTCACCGACAA | AGTGCACACAGCTCGACCTT | Primers for verifying *PiLLP* silencing |
| RTef1-F/R | ACTCCAAGAACGACCCTGCTAAGGCAACC | TTCGACGGCTCGAGGATGACCATGCAG | RT-PCR of *ef1* of *P. infestans* |
