# Supplementary material for: The Loricrin-Like Protein (LLP) of Phytophthora infestans Is Required for Oospore Formation and Plant Infection
Source: Front Plant Sci. 2017 Feb 9;8:142. doi: 10.3389/fpls.2017.00142 (PMC5298957; doi:10.3389/fpls.2017.00142)
Supplement: Table S3 — Percentage of aborted oospores of WT, CK, and transformants. [file Table3.DOCX]

**Table S3** Percentage of aborted oospores of WT, CK, and transformants

|  | Sample | No. of total oospores per disk | | No. of normal oospores | | No. of aborted oospores | | Percentage of aborted oospores (%) | | Average (%) | |
| --- | --- | --- | --- | --- | --- | --- | --- | --- | --- | --- | --- |
|  | WT | 992 |  | 958 |  | 34 |  | 3.4 |  | 4.0 |  |
|  |  | 990 |  | 945 |  | 45 |  | 4.5 |  |  |  |
|  |  | 1000 |  | 961 |  | 39 |  | 3.9 |  |  |  |
|  | CK | 1124 |  | 1100 |  | 24 |  | 2.1 |  | 2.3 |  |
|  |  | 1003 |  | 987 |  | 16 |  | 1.6 |  |  |  |
|  |  | 1009 |  | 978 |  | 31 |  | 3.1 |  |  |  |
|  | S3 | 613 |  | 324 |  | 289 |  | 47.1 |  | 47.4 |  |
|  |  | 656 |  | 346 |  | 310 |  | 47.3 |  |  |  |
|  |  | 580 |  | 302 |  | 278 |  | 47.9 |  |  |  |
|  | S16 | 356 |  | 189 |  | 167 |  | 46.9 |  | 45.6 |  |
|  |  | 304 |  | 168 |  | 136 |  | 44.7 |  |  |  |
|  |  | 284 |  | 156 |  | 128 |  | 45.1 |  |  |  |
|  | S84 | 566 |  | 298 |  | 268 |  | 47.3 |  | 47.9 |  |
|  |  | 595 |  | 308 |  | 287 |  | 48.2 |  |  |  |
|  |  | 553 |  | 287 |  | 266 |  | 48.1 |  |  |  |
|  | O36 | 1237 |  | 1203 |  | 34 |  | 2.7 |  | 2.6 |  |
|  |  | 1212 |  | 1180 |  | 32 |  | 2.6 |  |  |  |
|  |  | 1239 |  | 1210 |  | 29 |  | 2.3 |  |  |  |
|  | O38 | 1184 |  | 1158 |  | 26 |  | 2.2 |  | 2.5 |  |
|  |  | 1129 |  | 1098 |  | 31 |  | 2.7 |  |  |  |
|  |  | 1220 |  | 1187 |  | 33 |  | 2.7 |  |  |  |
